# Ganodermataceae—current status, research, and development in Lower Mekong Basin

**DOI:** 10.3389/fcimb.2025.1545135

**Published:** 2025-05-12

**Authors:** Samantha C. Karunarathna, Nimesha M. Patabendige, Thatsanee Luangharn, Kalani K. Hapuarachchi

**Affiliations:** ^1^ Center for Yunnan Plateau Biological Resources Protection and Utilization, College of Biology and Food Engineering, Qujing Normal University, Qujing, Yunnan, China; ^2^ School of Medical, Molecular and Forensic Sciences, Murdoch University, Perth, WA, Australia; ^3^ Center of Excellence in Fungal Research, Mae Fah Luang University, Chiang Rai, Thailand; ^4^ College of Biodiversity Conservation, Southwest Forestry University, Kunming, China

**Keywords:** Cambodia, *Ganoderma*, Laos, medicinal mushrooms, Thailand, Vietnam

## Abstract

The family Ganodermataceae encompasses several genera, including the widely studied *Ganoderma*, which is prominent in traditional medicine for its therapeutic properties. Species within this family, particularly *Ganoderma lucidum*, have been valued for centuries in regions such as China, Korea, and Japan for enhancing vitality, longevity, and overall health. However, the taxonomy of Ganodermataceae remains complex, with ongoing debates about species identification and classification. Members of this family are globally distributed, with the Lower Mekong Basin—comprising Laos, Thailand, Cambodia, and Vietnam—offering optimal conditions for their growth due to its warm, humid climate. In the Lower Mekong Basin, the species of Ganodermataceae are significant for their medicinal applications in treating conditions such as bronchitis, hepatitis, diabetes, and cancer. They also hold significant economic value, being used in products like teas, dietary supplements, and cosmetics. *Ganoderma lucidum* is particularly notable as a high-value market product in this region. Recent research has revealed a rich diversity of Ganodermataceae species in the region, highlighting their ecological roles, medicinal properties, and importance in plant pathology, particularly in addressing diseases in crops such as oil palm. These findings underscore the need for further research into the taxonomy, ecological functions, and potential applications of Ganodermataceae species. Advancing our understanding will support sustainable utilization, conservation efforts, and the maximization of their medicinal and commercial benefits.

## 1 Introduction

The Ganodermataceae family, encompassing the genus *Ganoderma*, has long been recognized for its diverse ecological, medicinal, and economic significance (Galappaththi et al., 2024; Karunarathna et al., 2024a). *Ganoderma* species, particularly *G. lucidum*, have played a central role in traditional medicine across Asia for centuries, where they are highly esteemed for their purported health benefits, including promoting vitality, longevity, and overall well-being (Karunarathna et al., 2024b; Klaus and Wan, 2024; Wu et al., 2024a). These fungi have been integral to various therapeutic practices in countries such as China, Korea, and Japan, where their use is deeply rooted in cultural and medicinal traditions (Chen et al., 2024; Zhong et al., 2024). The association of *Ganoderma* with health-enhancing properties, coupled with its economic value, has contributed to its widespread use in modern-day products such as dietary supplements, teas, and cosmetics (Karunarathna et al., 2024c; Wu et al., 2024b; Zheng et al., 2024). However, despite the long history of use and growing commercial importance of *Ganoderma*, its taxonomy remains complex and evolving, with ongoing debates surrounding species classification, identification, and ecological roles.

**Figure 1:**
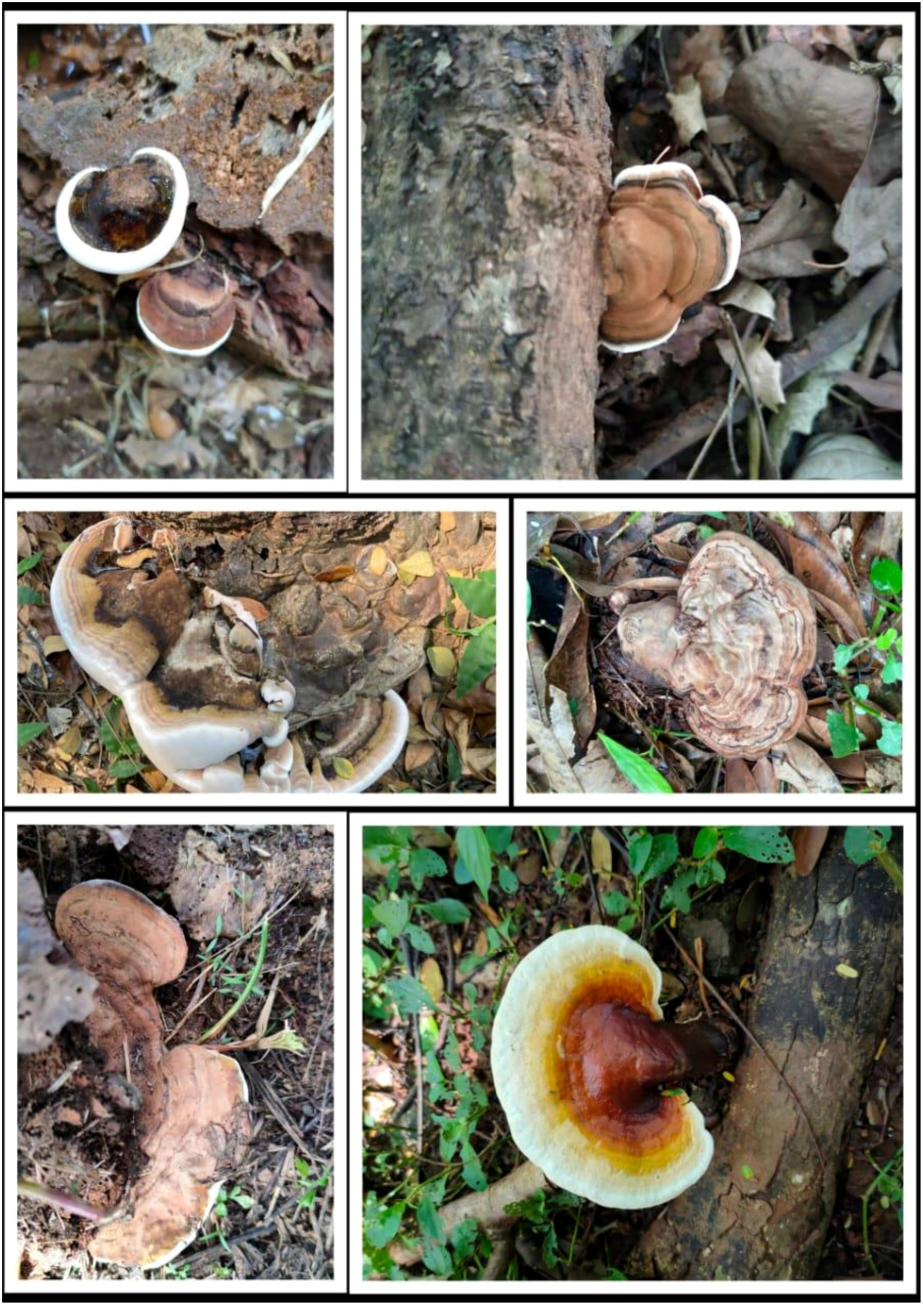
*Ganoderma* species found in Thailand.

*Ganoderma* species are found worldwide, thriving predominantly in tropical and subtropical climates, where their growth is facilitated by warm and humid conditions (Papp, 2019; Konara et al., 2024; Karunarathna et al., 2024d; Wei et al., 2024). These fungi are saprophytic or parasitic, often growing on decaying wood or living trees, and play a vital role in the decomposition of lignin and cellulose in forest ecosystems (Konara et al., 2022; Asad et al., 2024; Li et al., 2024). While the genus is distributed across a wide geographic range, the Lower Mekong Basin, which encompasses Laos, Thailand, Cambodia, and Vietnam, is a particularly notable region for the growth of *Ganoderma* species (Hapuarachchi et al., 2019a; Luangharn et al., 2019, 2021; Duong et al., 2022a; Pungpa et al., 2023, **Figure 1**). The warm, humid climate of the Lower Mekong Basin provides optimal conditions for the proliferation of these fungi, making it a key area for studying their diversity, ecological functions, and potential applications (Nguyen et al., 2023a; Wannasawang et al., 2023; Siriarchawatana et al., 2024).

The medicinal properties of *Ganoderma* are well documented in scientific literature, with numerous studies confirming its effectiveness in treating a range of ailments, including respiratory conditions such as bronchitis, liver diseases like hepatitis, metabolic disorders like diabetes, and even cancer (Chen et al., 2024; Lau et al., 2024; Rašeta et al., 2024). Bioactive compounds such as triterpenoids, polysaccharides, and peptidoglycans, which are found in various *Ganoderma* species, have been identified as responsible for many of these therapeutic effects (Rode et al., 2024; Xia et al., 2024). As a result, *Ganoderma* has become a focal point of research in the fields of pharmacology and natural product development (Azi et al., 2024; Wu et al., 2024b). The medicinal potential of the genus has led to the commercialization of *Ganoderma*-based products, which are widely consumed in the form of supplements, teas, and even incorporated into cosmetics (Karunarathna et al., 2024c; Thakur et al., 2024).

The economic significance of *Ganoderma* is particularly evident in the Lower Mekong Basin, where *G. lucidum* commands a high market value (Mortimer et al., 2014; Hapuarachchi et al., 2018a; Sopov et al., 2022; Nguyen et al., 2023a). In recent years, the demand for *Ganoderma*-derived products has seen a marked increase, driven by growing consumer interest in natural health solutions (Seethapathy et al., 2023; Ganoderma-Derived Products Market Overview, 2023; Ganoderma Market Trends and Growth Factors, 2024). This commercialization has spurred research into the cultivation, quality control, and standardization of *Ganoderma* products to meet market demand (Zhang et al., 2023; Wu et al., 2024b). Recent studies in the Lower Mekong Basin have emphasized the remarkable diversity of *Ganoderma* species and their ecological and medicinal importance (Nghien et al., 2019; Prasopthum et al., 2022; Nguyen et al., 2022, 2023; Wannasawang et al., 2023; Siriarchawatana et al., 2024). Researchers have identified several key species within the region and explored their roles in plant pathology, particularly in relation to diseases affecting economically important crops such as oil palm (Hapuarachchi et al., 2019a; Luangharn et al., 2021; Karunarathna et al., 2024d). These studies have underscored the genus’s dual role as both a beneficial medicinal organism and a potential plant pathogen, further complicating the classification and management of *Ganoderma* species (Nguyen et al., 2021; Suwannarach et al., 2022; Viet Hung et al., 2022; Thuy et al., 2023; Petwattanapha et al., 2024). The findings highlight the need for continued research into the ecological dynamics, taxonomy, and therapeutic potential of *Ganoderma* species in this region.

Furthermore, the increasing commercialization of *Ganoderma* products in the Lower Mekong Basin presents both opportunities and challenges. While the sustainable harvesting and cultivation of *Ganoderma* could provide economic benefits to local communities, the rising demand for these fungi also raises concerns regarding overexploitation, conservation, and the need for sustainable management practices. As research continues to uncover the complex interactions between *Ganoderma* species and their environment, it becomes clear that a comprehensive understanding of their taxonomy, ecological functions, and medicinal properties is essential for their sustainable utilization and conservation.

This paper aims to provide a comprehensive overview of the current status of *Ganoderma* research and development in the Lower Mekong Basin, with a particular focus on the diversity of species, their ecological roles, and their medicinal and economic significance. By examining the ongoing research efforts and highlighting key findings, this work seeks to contribute to the broader understanding of *Ganoderma* and its potential for future applications in medicine, agriculture, and commercial industries. Ultimately, a deeper understanding of *Ganoderma* will be essential for maximizing its benefits, ensuring its sustainable use, and addressing the challenges associated with its conservation and commercialization in the Lower Mekong Basin.

## 2 Taxonomy

The family *Ganodermataceae* is one of the primary families of polypores, comprising 15 accepted genera: *Amauroderma* s.str. Y.F. Sun, D.H. Costa, and B.K. Cui, *Amaurodermellus* Costa-Rezende, Drechsler-Santos & Góes-Neto, *Cristataspora* Robledo & Costa-Rezende, *Foraminispora* Robledo, Costa-Rezende & Drechsler-Santos, *Furtadoella* B.K. Cui & Y.F. Sun, *Furtadomyces* Leonardo-Silva, Cotrim & Xavier-Santos, *Ganoderma* P. Karst., *Haddowia* Steyaert, *Humphreya* Steyaert, *Magoderna* Steyaert, *Neoganoderma* B.K. Cui & Y.F. Sun, *Sanguinoderma* Y.F. Sun, D.H. Costa & B.K. Cui, *Sinoganoderma* B.K. Cui, J.H. Xing & Y.F. Sun, *Tomophagus* Murrill, and *Trachydermella* B.K. Cui & Y.F. Sun (Costa-Rezende et al., 2020; Sun et al., 2022a; Leonardo-Silva et al., 2022). Most species within the family are classified under the genus *Ganoderma*.

*Ganoderma* P. Karst. (Ganodermataceae, Agaricomycetes) was first described by Karsten (1881), based on *Polyporus lucidus* (Curtis) Fr., to encompass species characterized as laccate and stipitate white rot fungi. It is the most prolific genus within the family, with 498 species recorded in Index Fungorum (2025) (http://www.indexfungorum.org/) and 542 in MycoBank (http://www.mycobank.org/), as of 16 March 2025. The genus *Ganoderma* is defined by its laccate or non-laccate basidiocarps, sessile to stipitate basidiomata, white to pale yellow margins, and red-brown, truncate, double-walled basidiospores. These spores feature an apical germinal pore, a thin, colorless external wall (exosporium), and brown to dark brown interwall pillars (endosporium) (Hapuarachchi et al., 2019a; Sun et al., 2022a; He et al., 2022, 2024). The double-walled basidiospores with interwall pillars are a key diagnostic feature for the genus (Wei et al., 2024). Species within this genus exhibit diverse characteristics, including variations in the shape and color of the fruiting body, host specificity, and geographical distribution, which aid in species identification (Li et al., 2023; Mardones et al., 2023; Cho et al., 2024; Ndeh et al., 2024). However, the species concept in *Ganoderma* remains neither widely agreed upon nor clearly defined due to significant morphological variability, even within the same species (Hapuarachchi et al., 2019b; Costa-Rezende et al., 2020; Pristas et al., 2023). Environmental factors, inter-hybridization, and morphological biases further complicate the identification of *Ganoderma* species (Luangharn et al., 2023). The genus also presents considerable taxonomic challenges, as its morphology varies significantly across environments, while microscopic features remain consistent (Cortina-Escribano et al., 2024). Nearly half of the recorded entries have been identified as synonyms, underscoring the complexity of *Ganoderma* taxonomy (He et al., 2022; Galappaththi et al., 2024). Nevertheless, robust molecular and phylogenetic analyses have confirmed the existence of 191 valid *Ganoderma* taxa (Sun et al., 2022b; Cabarroi-Hernández et al., 2023; He et al., 2024). *Ganoderma* species are also known for causing white rot in woody plants and are further valued for their medicinal properties (Kumari et al., 2024).

*Amauroderma* s.str. Y.F. Sun, D.H. Costa, and B.K. Cui, the second largest genus within Ganodermataceae, is distinguished by a di-trimitic hyphal system and an endospore wall with solid columnar to semi-reticulated ornamentation (Costa-Rezende et al., 2017; Sun et al., 2020). The genus is distributed mainly in the Neotropics and tropical or subtropical areas of Africa, Asia, and Oceania (Costa-Rezende et al., 2016; Peres et al., 2023). *Amauroderma* contains sterols, flavonoids, fatty acids, polysaccharides, and triterpenes, offering antioxidant, anti-inflammatory, neuroprotective, and antibacterial effects. Its bioactive components show therapeutic potential, especially for age-related diseases (Hapuarachchi et al., 2018b). *Sanguinoderma* (Blume and T. Nees) Y.F. Sun, D.H., Costa & B.K. Cui, one of the largest genera in Ganodermataceae after *Ganoderma* and *Amauroderma*, is distinguished by annual basidiomata with a corky to woody texture, a central or lateral stipe, and a nearly sessile pileus. The pileus, suborbicular to reniform, may be glabrous or tomentose, with concentric zones or radial furrows. Its pore surface turns blood red when bruised (Sun et al., 2020). *Sanguinoderma* includes species with notable medicinal and cultural significance. Most species in the genus inhabit soil and are primarily distributed across tropical and subtropical regions, including Africa, Asia, North America, Oceania, and South America (Sun et al., 2020, 2022a; Niu et al., 2024).

## 3 Diversity of Ganodermataceae in Lower Mekong Basin

The Lower Mekong River Basin is divided into four geographic regions, covering a catchment area of approximately 571,000 km². This extensive basin encompasses much of northeastern Thailand, nearly all of Lao PDR and Cambodia, and the southern tip of Vietnam (Mekong River Commission, accessed 16 March 2025). The Mekong River is essential for over 245 million people in the Lower Mekong Region, supporting agriculture, fisheries, food security, and economic stability. However, hydropower projects threaten the resources of the river by altering water flow, disrupting fish migration, and affecting agriculture, which could jeopardize the region’s economy (International Rivers, 2021). Mushroom growing is increasingly becoming popular in this region as a means to generate income, improve the quality of life for rural people, and promote sustainable development in local communities. *Ganoderma lucidum* has a 33.0–50.0 US$ market price per 1 kg as a commercial mushroom in Thailand (Tippayawong et al., 2011).

*Ganoderma japonicum* Imaz is recognized as a medicinal mushroom, known for its potential therapeutic benefits (Chandrasrikul et al., 1978). The disease of oil palm (*Elaeis guineensis* Jacq.) caused by *G. boninense* becomes the most important disease in Thailand, Indonesia, and Malaysia. Chandrasrikul et al. (1986) reported these groups of fungi had not been fully investigated and described in Thailand, and some species of *Ganoderma* and allied genera are considered possible parasites of living trees, found on dead trees, logs, and stumps and known to have some medicinal value. Furthermore, he studied a collection of seventeen specimens, described and identified by comparing morphological characters. They were *G. lucidum* (Leyss. ex Fr.) Karst. *G. colossum* (Fr.) Bres. *G. applanatum* (Fr.) Karst. and *Amauroderma rugosum* (Nees) Bose. Ganodermataceae family members: *Amauroderma rugosum* (Fr.) Torr. *Ganoderma applanatum* (Fr.) Pat., *G. australe* (Fr.) Pat., and *G. lucidum* (Fr.) Karst. were found in Northern Thailand (Hjortstam and Ryvarden, 1982).

The diversity of mushrooms surveyed in the Plant Genetic Protection Area of RSPG, Nampung Dam EGAT, Sakhon Nakhon Province in Thailand during the rainy season between July and August discovered *Amauroderma rugosum* (Blume et Nees) Bres., *Ganoderma applanatum* (Pers. Ex Wallr.) Pat., *Ganoderma chiungchungense* X.L.Wu *Ganoderma dahlii* (Henn.) Aoshima, and *G. lucidum* (Leys.ex Fr.) Karst (Pitakpong et al., 2013). *Ganoderma subresinosum* Fr. and two unidentified *Ganoderma* species were reported from the Pattani watershed in Southern Thailand (Rangpan, 2015); furthermore, he found *Amauroderma dubiopansum* and *Amauroderma rude* (B.) Pat from the same location.

The Ganodermataceae was studied in Son Tra, Danang City, Vietnam, where 38 species across three genera (*Ganoderma*, *Amauroderma*, and *Haddowia*) were identified. *Ganoderma* had the highest species richness (29 species), followed by *Amauroderma* (8 species) and *Haddowia* (1 species) (Phu, 2023). Mushroom surveys in Cambodia identified 1,383 specimens, including *Ganoderma* species, which accounted for 5.6% of the collected fungi. Specimens were collected from western (Koh Kong forests) and eastern (Seima and Mondulkiri forests) regions, covering elevations from 0 to 750 m. *Ganoderma* sp. was among the dominant genera in both regions, highlighting its widespread distribution. The biodiversity analysis revealed 238 species across various elevations, with higher diversity observed at 251–500 m. These findings emphasize the significance of *Ganoderma* in Cambodia’s fungal biodiversity and its potential for ecological and economic applications (Kim et al., 2016; Wijaya et al., 2021). In addition, species of Ganodermataceae found in the Lower Mekong Basin, along with their current legitimate names, are listed in **Table 1**.

**Table 1:** Species of Ganodermataceae found in the region.

Species name	Country	Reference
*Ganoderma adspersum*	Thailand, Laos	Hapuarachchi et al., 2019a; Luangharn et al., 2021
*G. amboinense*	Vietnam	Kiet, 1998
*G. applanatum*	Thailand, Laos, Vietnam, Cambodia	Pham, 1961; Chandrasrikul et al., 1986; Hjortstam and Ryvarden, 1982; Chalermpongse, 1989; Chalermpongse, 1991a; Kiet, 1998; Cong, 2010; Pitakpong et al., 2013; Rangpan, 2015; Kim et al., 2017; Van Trung et al., 2018; Hapuarachchi et al., 2019a; Luangharn et al., 2021
*G. australe*	Thailand, Laos, Vietnam	Chalermpongse, 1989, 1999; Kiet, 1998; Rungjindamai et al., 2008; Tompong and Kunasakdakul, 2014; Keomanykham et al., 2016; Hapuarachchi et al., 2019a; Luangharn et al., 2017; Hung et al., 2020; Wongkhieo et al., 2023
*G. balabacense* (= *G. chalceum*)	Vietnam	Kiet, 1998
*G. boninense*	Thailand, Vietnam	Chandrasrikul et al., 1978; Kiet, 1998; Rungjindamai et al., 2008
*G. casuarinicola*	Thailand	Luangharn et al., 2019, 2021
*G. capense*	Vietnam	Kiet, 1998
*G. chalceum*	Cambodia	Kim et al., 2017
*G. chiungchungense*	Thailand	Pitakpong et al., 2013
*G. cochlear*	Vietnam	Kiet, 1998
*G. colossum*	Thailand, Vietnam	Chalermpongse, 1989; Chandrasrikul et al., 1986; Kiet, 1998; Kleinwächter et al., 2001; El Dine et al., 2009
*G. dahlii*	Thailand, Laos	Pitakpong et al., 2013; Lee et al., 2021
*G. donkii*	Thailand	Hapuarachchi et al., 2019a
*G. ellipsoideum*	Thailand	Luangharn et al., 2021
*G. flexipes*	Laos, Vietnam	Kiet, 1998; Hapuarachchi et al., 2019a; Luangharn et al., 2019, 2021
*G. gibbosum*	Laos, Thailand, Vietnam	Kiet, 1998; Hapuarachchi et al., 2019a; Luangharn et al., 2021, 2023
*G. hochiminhensis*	Vietnam	Luangharn et al., 2021
*G. japonicum* (= *G. lucidum*)	Thailand	Chandrasrikul et al., 1978
*G. lobatum*	Vietnam	Kiet, 1998
*G. laccatum* (= *G. lucidum*)	Vietnam	Kiet, 1998
*G. lingzhi*	Laos	Hapuarachchi et al., 2019a
*G. lucidum*	Thailand, Vietnam, Cambodia	Chandrasrikul et al., 1986; Hjortstam and Ryvarden, 1982; Kiet, 1998; Chalermpongse, 1999; Rungjindamai et al., 2008; Hung and Nhi, 2012; Rangpan, 2015; Kim et al., 2017; Truyen and Patacsil, 2017; Hawkeswood et al., 2020; Luangharn et al., 2021; Nguyen et al., 2021; Duong et al., 2022b
*G. luteomarginatum*	Laos	Hapuarachchi et al., 2019a
*G. mastoporum* (= *G. orbiforme*)	Vietnam	Thang et al., 2013
*G. mirabile*	Vietnam	Phúc et al., 2014
*G. multipileum*	Thailand, Vietnam, Laos	Pedersen and Thammavong, 2016; Luangharn et al., 2021; Nguyen et al., 2023b; Duong et al., 2022b; Truyen and Patacsil, 2017
*G. multiplicatum*	Vietnam	Nguyen et al., 2023c
*G. nasalaense*	Laos	Hapuarachchi et al., 2019a
*G. neojaponicum*	Laos, Cambodia, Vietnam	Hien et al., 2014; Kim et al., 2017; Hapuarachchi et al., 2019a
*G. oerstedii*	Vietnam	Kiet, 1998
*G. orbiforme*	Thailand, Laos, Cambodia	Kim et al., 2017; Hapuarachchi et al., 2019a; Luangharn et al., 2021; Wannasawang et al., 2023
*G. orofravum*	Vietnam	Kiet, 1998
*G. ostracodes*	Vietnam	Kiet, 1998
*G*. *petchii*	Vietnam	Kiet, 1998
*G. philippi*	Thailand, Vietnam	Kiet, 1998; Luangharn et al., 2021
*G. pfeifferi*	Vietnam	Kuo et al., 2016
*G. ramosissimum*	Vietnam	Kiet, 1998
*G. resinaceum*	Cambodia	Kim et al., 2017
*G. sichuanense*	Thailand, Laos	Pedersen and Thammavong, 2014; Thawthong et al., 2017; Luangharn et al., 2021
*G. sinense*	Thailand, Vietnam	Luangharn et al., 2021; Nguyen et al., 2023a
*G. subresinosum*	Thailand, Laos, Vietnam	Rangpan, 2015; Truyen and Patacsil, 2017; Hapuarachchi et al., 2019a; Luangharn et al., 2021; Duong et al., 2022a
*G. tenue*	Vietnam	Kiet, 1998
*G. testaceum*	Vietnam	Kiet, 1998
*G. thailandicum*	Thailand	Luangharn et al., 2019, 2021
*G. tornatum*	Vietnam	Kiet, 1998
*G. tropicum*	Thailand, Laos	Kiet, 1998; Hapuarachchi et al., 2019a; Luangharn et al., 2019, 2021, 2023
*G. tsugae*	Thailand, Vietnam	Nguyen et al., 2021; Naksuwankul et al., 2022
*G. williamsianum*	Thailand	Surawut et al., 2023
*G. xanthocreas*	Vietnam	Kiet, 1998
*Amauroderma auriscalpium*	Vietnam	Kiet, 1998
*Amauroderma bataanense* (= *Sanguinoderma bataanense*)	Vietnam	Kiet, 1998
*A. dubiopansum* (= *Foraminispora rugosa*)	Thailand	Rangpan, 2015
*A. elmerianum* (= *Sanguinoderma elmerianum*)	Vietnam	Kiet, 1998
*A. preussi* (= *Sanguinoderma preussi*)	Laos	Hapuarachchi et al., 2018b
*A. pullatum* (= *Sanguinoderma rude*	Vietnam	Kiet, 1998
*A. rude* (= *Sanguinoderma rude*)	Thailand, Laos, Cambodia, Vietnam	Rangpan, 2015; Kim et al., 2017; Kiet, 1998
*Amauroderma rugosum* (= *Sanguinoderma rugosum*)	Thailand, Laos, Cambodia, Vietnam	Chandrasrikul et al., 1986; Hjortstam and Ryvarden, 1982; Pitakpong et al., 2013; Kim et al., 2017; Truyen and Patacsil, 2017; Hapuarachchi et al., 2018b; Lee et al., 2021; Surawut et al., 2023
*A. salebrosum* (= *Sanguinoderma preussi*)	Vietnam	Kiet, 1998
*A. schomburgkii*	Laos	Hapuarachchi et al., 2019a
*A. scopulosum*	Vietnam	Kiet, 1998
*A. subrugosum*	Vietnam	Kiet, 1998
*A. subresinosum* (= *G. subresinosum*)	Vietnam	Tham et al., 2006
*A.cf. yunnanense* (=*Foraminispora yunnanense*)	Vietnam	Kiet, 1998
*Haddowia longipes*	Laos	Hapuarachchi et al., 2019a
*Humphreya endertii*	Vietnam	Tham et al., 2018
*H. laccocoffeatum*	Vietnam	Tham et al., 2018
*Tomophagus cattienensis*	Vietnam	Le et al., 2016

N/A (Not available).

## 4 Cultivation of *Ganoderma* and other species from related genera in the Lower Mekong Basin

### 4.1 Thailand

Thailand plays a vital role in the Lower Mekong Basin, standing out for its advanced agricultural practices and rich biodiversity, which provide ideal conditions for mushroom cultivation. With a long history of mushroom farming, the Thai government has actively promoted this sector to improve rural livelihoods. Initiatives such as the Royal Mushroom Projects and government-supported loan programs have significantly contributed to steady growth in mushroom production. These efforts showcase Thailand’s commitment to sustainable agriculture and rural development, serving as a model for neighboring countries in the region (Kwon and Thatithatgoon, 2004). *Ganoderma* was cultivated for a long time in Thailand; however, the information for their activities and chemical components was insufficient (Poomsing et al., 2013). *Ganoderma applanatum*, *G. australe*, *G. curtisii*, *G. lucidum*, *G. oregonense*, *G. orbiforme, G. resinaceum*, G. *sichuanense*, *G. sinense*, *G. tenus*, *G. tropicum*, and *G. tsugae* are presently cultivated in Thailand (Pothisuwan et al., 2010; Thawthong et al., 2014; Luangharn et al., 2017, 2019; Wannasawang et al., 2023).

*Ganoderma australe*, identified from Thailand, grew best at 25°C–30°C and pH 7–8, with sorghum and barley as the top grain media for spawn production. Potato Dextrose Agar (PDA) was ideal for mycelial growth. Cultivated on para rubber sawdust with additives, mycelia spread fully after 18 days at 30°C and 60%–75% humidity. Three fruiting cycles yielded decreasing mushroom weights (Luangharn et al., 2017). Thai *G. lucidum* (G2) has been grown in Thailand as part of the Royal Project since 1988 (Petcharat, 1996). The production of *G. lucidum* and its spores was studied at the Muang Ngai Special Agricultural Project under the patronage of Her Majesty Queen Sirikit in the fiscal year 2009. The findings indicated the potential for commercial production of these mushrooms and spores. In the Thai markets, dried *G. lucidum* is priced at 850 baht (USD 25) per kilogram, while spores ranged from 2,000 to 100,000 baht (USD 59 to 2,957) per kilogram. However, if farmers are to adopt *Ganoderma* cultivation, they must adhere to good agricultural practices to ensure effective production (Pothisuwan et al., 2010). Furthermore, substitution of sawdust with Nash leaves (*Vetiveria zizaniodes* L.) can reduce the production cost of *G. lucidum* (Sornprasert and Aroonsrimorakotet, 2014). Sugarcane bagasse was evaluated as a substrate for cultivating *G. lucidum* in comparison to sawdust. While *G. lucidum* grew faster in sawdust, sugarcane bagasse resulted in higher cellulase activity and biological efficiency. These findings suggest sugarcane bagasse is a promising alternative substrate for *G. lucidum* production, supporting improved enzyme activity and yield (Ninluam et al., 2016). *Ganoderma lucidum* strains (GA1, GA2, and GA3) cultivated in Tam Dao, Vietnam, exhibited similar polysaccharide levels, with GA3 showing the highest lucidenic N acid (0.33 mg/g) and ganoderic acid (2.38 mg/g), while GA1 had the highest ganodermanontriol content (0.3 mg/g) (Nghien et al., 2019). The isolation of strong *G. lucidum* mycelium is crucial for successful transplantation. Mycelium was cultivated on PDA, brown rice, and grain substrates with varying ratios of rice bran, MgSO_4_, and CaCO_3_. Using wheat bran supplemented with 4 g of rice bran and MgSO_4_ at different concentrations significantly enhanced *G. lucidum* mycelial growth on *Manihot esculenta* substrate (Nhung, 2019). *Ganoderma lucidum* yields the highest polysaccharide content when grown under specific conditions. The optimal growing environment includes temperatures of 25°C–30°C and humidity of 60%–70% for the first 34 days, followed by 22°C–28°C and 80%–90% humidity for the next 33 days, and finishing at 22°C–28°C with 60%–70% humidity. The best extraction conditions involve a 1:40 mushroom/solvent ratio, 80°C temperature, 90-min extraction time, and three extractions. These conditions maximize polysaccharide content for medicinal use (Duyen and Mai, 2023). Wild *Ganoderma* strains from northern Thailand, including *G. sichuanense* and *G. orbiforme*, were studied for optimal growth conditions at 25°C–30°C and pH 4–8. *Ganoderma sichuanense* thrived on potato sucrose agar and *G. orbiforme* on oatmeal agar. Both species produced fruiting bodies in bag culture, with *G. orbiforme* also thriving in field conditions (Wannasawang et al., 2023). The growth and bioactive compound production in *G. sichuanense* was optimized using various fruit peels as substrates. Durian peel was most effective for mycelial growth on solid media, achieving 9.4 mm/day. In liquid culture, mango, durian, and mangosteen peels resulted in similar mycelial yields (around 11 g/L). Durian peel (0.1% concentration) significantly boosted polysaccharide (74.25 mg/g), phenolic content (57.26 mg GAE/g), and triterpenoid production (21.52 mg/g) after 21 days. Using fruit peels as supplements for cultivation enhances bioactive compound production (Apiwatanapiwat et al., 2024). *Hed Nua Yang* (*G. subresinosum* Fr.), an edible medicinal mushroom from Thailand, grows best on PDA at 25°C and pH 5.7. Cultivation on sawdust supplemented with gypsum, rice bran, and magnesium sulfate produced mycelial growth in 15 days, with fruiting beginning after 4 months and yielding 11.4 g per bag in the first flush (Petcharat, 1996).

*Ganoderma tropicum* is reported for the first time from Chiang Rai Province, Thailand, and its optimal conditions for mycelial growth were found on PDA, MEA, and YPD media at pH 7–8 and temperatures of 25°C–28°C. Although successful growth conditions were identified, fruiting could not be achieved, making this the first report on the mycelial growth of this species (Luangharn et al., 2019). Green mold disease, caused by *Trichoderma* species, poses a major threat to *Ganoderma* cultivation. Recently, an outbreak in a *Ganoderma* farm in Songkhla, Thailand, prompted research to identify the causative *Trichoderma* species, and it was identified that *T. harzianum*, *T. pleuroticola*, and *T. reesei*. This study marks the first report of *T. pleuroticola* and *T. reesei* causing green mold disease in *G. lingzhi* in Thailand (Ubolsuk and Pornsuriya, 2022).

### 4.2 Vietnam

The rising demand for *Ganoderma* sp. in Vietnam highlights the need for better breeding programs hindered by limited genetic knowledge. A study was analyzed nine accessions from southern Vietnam using morphological and molecular methods with 20 ISSR (Inter Simple Sequence Repeat) markers, revealing significant variation in growth, fruiting bodies, and genetic composition. Two main genetic groups were identified, offering insights for classification and breeding improvements (Vo and Le, 2019). The effects of vanadium (V), selenium (Se), and germanium (Ge) on *G. lucidum* mycelia were examined. Se and V showed high toxicity in pure culture, whereas Ge was non-toxic at tested levels. In cultivation, *G. lucidum* grown on V-enriched sawdust developed mature fruit bodies with low V bioaccumulation. Se was effectively absorbed and concentrated in the pileus, with depletion via basidiospores. Ge was readily absorbed and transported into fruit bodies (Le Tham et al., 1999). The biological efficiency and bioactive components of three *G. lucidum* strains (GA1, GA2, and GA3) cultivated in Tam Dao, Vietnam, were evaluated. All strains grew well on rice-bran-supplemented PDA, colonized in 9 days, and yielded 13%–17%, making them suitable for commercial cultivation (Nghien et al., 2019). Optimal conditions for *G. lucidum* strain GA3 were identified as potato, glucose, and agar (PGA) media supplemented with rice bran, a temperature range of 25°C–30°C for mycelial growth, and a pH range of 4–12. For fruiting body development, the best substrate consisted of 87% sawdust, 4% corn powder, 8% rice bran, and 1% calcium carbonate (CaCO_3_) (Nguyen et al., 2019). An IoT-based monitoring system for indoor *G. lucidum* cultivation was developed to track temperature and humidity in real time, optimizing growth conditions. The system proved cost-effective and practical, with results showing it successfully maintained optimal parameters. The produced *G. lucidum* met Vietnamese regulatory quality standards (Nguyen et al., 2023d). A study found glucose and ammonium sulfate to be the best carbon and nitrogen sources for its mycelial growth (5.52–5.63 mm/day). Ga-TB grows well at pH 5.0–10.0 (optimal at 7.0) and temperatures of 25°C–30°C (6.03–6.16 mm/day) (Nguyen et al., 2023e). *Ganoderma sinense* was successfully cultivated under optimized conditions in Vietnam. It is identified fructose (15 g/l) and yeast extract (1 g/l) as the best carbon and nitrogen sources, with optimal pH 7 and temperature 25°C–30°C for mycelial growth. The fastest growth occurred with a substrate mix of 69% rice grains, 30% sawdust, and 1% calcium carbonate, while the highest fruiting body yield (2.95% biological efficiency) was achieved with 96% sawdust, 1% wheat bran, and 1% lime. These findings highlight the potential for commercial cultivation of *G. sinense* strain GA21 (Nguyen et al., 2023). *Ganoderma lucidum* strain Ga-TB stands out for its high yield in Vietnam.

### 4.3 Cambodia

Cost-effective local substrates in Cambodia, including rubber tree sawdust, sugarcane bagasse, and acacia sawdust, were tested for thermophilic mushrooms, including *Pleurotus sajor-caju*, *G. lucidum*, *Auricularia auricula*, and *Lentinula edodes*. Rubber tree sawdust and sugarcane bagasse showed high efficiency (~60%), while acacia sawdust, although less efficient (22.4%), was 6.5 times cheaper, making it a viable option to reduce production costs in rural areas (Chang et al., 2016). Another study on mushroom cultivation in Cambodia highlights the production of *G. lucidum* and its specific requirements for successful growth. *Ganoderma lucidum* thrives at 18°C–25°C and 85%–90% humidity, with sawdust substrates yielding higher biological efficiency and fruiting body production compared to log cultures. Contamination rates were lower in bag cultures (13 cases) than in logs (22 cases). These findings emphasize the importance of optimizing substrates and growing conditions for *G. lucidum* to enhance its production in the mushroom industry in Cambodia (Srey, 2019). **Table 2** presents a summary of the important findings regarding the growth and production of bioactive compounds in different *Ganoderma* species within the Lower Mekong Basin.

**Table 2:** Summary of key findings related to the growth and bioactive compound production of various *Ganoderma* species in Lower Mekong Basin.

Country	Species	Key findings	References
Thailand	*G. applanatum, G. australe, G. curtisii, G. lucidum, G. oregonense, G. orbiforme, G. resinaceum, G. sichuanense, G. sinense, G. tenus, G. tropicum, G. tsugae*	*G. australe* grows best at 25°C–30°C, pH 7–8, with sorghum and barley as top grain media, sawdust substitution with Nash leaves lowers production cost. - Sugarcane bagasse improves enzyme activity and yield. - Wild *G. sichuanense* and *G. orbiforme* studied for optimal growth. - Fruit peel substrates enhance *G. sichuanense* bioactive compound production	Kwon and Thatithatgoon, 2004; Poomsing et al., 2013; Pothisuwan et al., 2010; Thawthong et al., 2014; Luangharn et al., 2017, 2019; Wannasawang et al., 2023; Luangharn et al., 2017; Petcharat, 1996; Ninluam et al., 2016; Sornprasert and Aroonsrimorakotet, 2013; Apiwatanapiwat et al., 2024; Ubolsuk and Pornsuriya, 2022
Vietnam	*G. lucidum, G. sinense*	ISSR markers identified two main genetic groups. - *G. lucidum* optimal mycelial growth: PGA media, 25°C–30°C, pH 4–12. - Fruiting body substrate: 87% sawdust, 4% corn powder, 8% rice bran, 1% CaCO_3_. - IoT-based monitoring system optimized *G. lucidum* growth. - *G. sinense* commercialized with optimal conditions: 15 g/L fructose, 1 g/L yeast extract, pH 7, 25°C–30°C.	Vo and Le, 2019; Le Tham et al., 1999; Nghien et al., 2019; Nguyen et al., 2019, 2023; Duyen and Mai, 2023
Cambodia	*G. lucidum*	- Rubber tree sawdust and sugarcane bagasse showed high efficiency (~60%). - *G. lucidum* grows best at 18°C–25°C, 85%–90% humidity. - Sawdust substrates improved biological efficiency compared to log cultures. - Contamination rates lower in bag cultures (13 cases) than logs (22 cases).	Chang et al., 2016; Srey, 2019

## 5 *Ganoderma* as a plant pathogen

The Lower Mekong Region, comprising Thailand, Laos, Cambodia, and Vietnam, faces significant agricultural challenges due to pathogenic *Ganoderma* species. Diseases such as basal stem rot (BSR) in oil palms and root and butt rots in economically and ecologically valuable trees are prevalent across the region. In addition to Thailand, where *G. boninense*, *G. applanatum*, and *G. lucidum* have been well documented, similar infections impact crops and forests in neighboring countries. Research on biodiversity and innovative biocontrol strategies is vital for sustainable management in the region.

### 5.1 *Ganoderma*-associated diseases and prevention methods

#### 5.1.1 *Ganoderma applanatum*, *G. australe*, and *G. colossum*

*Ganoderma* trunk rot has been identified as a major disease affecting oil palm (*Elaeis guineensis*) plantations in the southern provinces of Thailand (Limsrivilai et al., 1980; Watanavanich, 1982). Since 1977, dangerous forest tree diseases in Thailand, such as butt and heart rots, were caused by *G. applanatum*, *G. australe*, and *G. colossum* (Chalermpongse, 1989; Likhitekaraj and Tummakate, 2000). Research conducted between 1993 and 1997 at the Mae Klong Watershed Research Station in Thong Phaphoom District, Kanchanaburi Province, Western Thailand, investigated the biodiversity dynamics of ectomycorrhizal (ECM) and wood-rotting fungi. This study identified *G. australe* and *G. lucidum* as a prominent species of Basidiomycota (Chalermpongse, 1999). Four *Ganoderma* species were documented, including *G. australe* from Aung-Reu-Nai Wildlife Sanctuary, along with two additional unidentified *Ganodermataceae* species recorded in the upper Khao Soi Dao Wildlife Sanctuary, in Eastern Thailand (Klingesorn, 1998a, 1998b). In addition, species of *Ganoderma* have been reported from Nong-rawieng Plant Genetics Forest, Nakhon Ratchasima, Thailand (Rodtong et al., 1998).

#### 5.1.2 Ganoderma boninense

A 2-year study of aphyllophoraceous fungi in Thai forests included *Amauroderma parasiticum* Corner, Beih., and *G. boninense* in the checklist compiled by Choeyklin et al. (2011). *Ganoderma boninense* has been identified as one of the major pathogens responsible for BSR in oil palm plantations in Southern Thailand (Pornsuriya et al., 2013). Wilting associated with BSR is recognized as the most devastating disease affecting oil palm, with the pathogen typically found growing at the basal portion of infected palms (Lim et al., 1992).

#### 5.1.3 Ganoderma lucidum

*Ganoderma lucidum* was also associated with butt rot in mangrove tree species at different mangrove localities in southern Thailand (Chalermpongse, 1991b).

#### 5.1.4 Ganoderma pseudoferreum

Red root disease caused by *G. pseudoferreum* was discovered in rubber plantations in the Surat Thani and Chumporn provinces (Panchan et al., 1995). Two root rot diseases impacting rubber plantations in Thailand include white root disease, caused by *Rigidoporus lignosus*, and red root disease, caused by *G. pseudoferreum*. *Ganoderma pseudoferreum* infects trees through the roots, spreading to the trunk and producing dark red mycelium with light brown edges. Preventive treatments with BERET 400 FS (fenpiclonil) and SCORE 250 EC (difenoconazole) were effective when applied twice annually. Curative treatments also controlled the disease effectively, provided infections were under 25% of the trunk circumference (Panchan et al., 1995).

### 5.2 Disease prevention and management of *G. boninense*

#### 5.2.1 Biological control agents

##### 5.2.1.1 Fungal and actinomycete-based biocontrol

A biological control agent, *Chaetomium* species, may release antagonistic substances to suppress *G. boninense*, a pathogen that causes significant losses in palm oil production (Soytong, 2014). Actinomycetes isolated from the oil palm rhizosphere were tested for their ability to inhibit *G. boninense*. Three species—*Streptomyces abikoensis, Kitasatospora nipponensis*, and *S. angustmyceticus*—were identified as effective inhibitors (Pithakkit et al., 2015).

##### 5.2.1.2 Potent biocontrol strains and their bioactive compounds

*Streptomyces palmae* CMU-AB204T was identified as a potent biocontrol agent, reducing BSR severity by over 75% and enhancing plant vigor in oil palm seedlings. Bioactive compounds from *S. palmae*—actinopyrone A, anguinomycin A, and leptomycin A—showed strong anti-*Ganoderma* activity, highlighting its potential as a protective agent for oil palm (Sujarit et al., 2020). The use of palm oil mill effluent (POME) for producing antifungal compounds by *Streptomyces philanthi* RM-1-138 demonstrated potential in inhibiting *G. boninense*, *Ceratocystis paradoxa*, and *Curvularia oryzae*. *In-vitro* tests revealed high inhibition of these fungal pathogens, with POME offering optimal conditions for compound production. The antifungal compounds exhibited strong activity against these oil palm pathogens, suggesting the potential of POME as a sustainable resource for biocontrol in oil palm disease management (Boukaew et al., 2022).

##### 5.2.1.3 *Trichoderma*-based biocontrol strategies

The biocontrol agent *Trichoderma virens* K1-02 demonstrated effective suppression of *G. boninense* through volatile antifungal compounds and enzyme production (Anothai et al., 2023). Greenhouse trials showed treated oil palm roots had higher lignocellulose content, offering a promising strategy for BSR management (Anothai and Chairin, 2024). The effects of *G. boninense* infection and *Trichoderma asperellum* T76-14 treatment on oil palm seedlings were investigated. *Ganoderma* increased phenylalanine ammonia-lyase (PAL) activity early, while *T. asperellum* enhanced PAL and polyphenol oxidase (PPO) activity. *Trichoderma*-treated seedlings showed no visible BSR symptoms after 20 weeks and reduced necrosis compared to controls. Morphological traits were largely unaffected, highlighting the potential of *T. asperellum* as an early-stage biocontrol for BSR (Samlikamnoed et al., 2023).

##### 5.2.1.4 Microbial diversity and disease suppression

Asymptomatic trees in *G. boninense*-infected oil palm plantations exhibit higher microbial diversity and a greater abundance of beneficial bacteria like *Actinobacteria* and *Firmicutes* compared to symptomatic trees. These bacteria are linked to disease suppression and plant health (Anothai and Chairin, 2022).

#### 5.2.2 Chemical and microbial control strategies

Plant extracts, antagonistic microorganisms, and fungicides were evaluated for controlling *G. boninense*. Among the plant extracts, *Carica papaya* showed the highest inhibition at 41.26%. Antagonistic bacterial isolates B001, B002, and B003, as well as fungal isolate T003, demonstrated significant efficacy. Fungicides such as prochloraz, kresoxim-methyl, and chlorothalonil showed mycelium growth suppression of up to 98.96% (Chaisit et al., 2017). The effects of micronutrients (Fe, Zn, Mo, and Mn at 1 mM) on ligninolytic enzymes produced by *G. boninense* on oil palm root substrates were evaluated. Zn and Mn enhanced laccase activity in solid-state cultures, while all tested micronutrients significantly reduced activities of lignin peroxidase (LiP), manganese peroxidase (MnP), and laccase in crude extracts (Intara-Anun and Chairin, 2021).

#### 5.2.3 Environmental and agronomic factors influencing disease control

In Thailand, the relationship between soil properties, fungal enzymes, and plant defense responses in *G. boninense*–infected oil palm plantations highlight that organic matter and nutrients enhance defenses like PAL and chitinase, while fungal enzymes correlate with organic carbon and low soil pH, collectively influencing BSR disease (Anothai and Chairin, 2020). Research identified key factors, including temperature, potassium, boron, and mancozeb, that inhibit *G. boninense* growth and lignocellulosic enzyme activity. The effects of temperature and light on *G. boninense* enzyme activities were studied under laboratory conditions. Higher temperatures (35°C–40°C) and light exposure reduced laccase, lignin peroxidase, and manganese peroxidase activities. These results suggest that temperature and light can be utilized in future strategies for managing BSR disease in oil palm (Intara-Anun and Chairin, 2023).

#### 5.2.3 Genetic and technological advances in disease management

Hyperspectral and multispectral remote sensing effectively detected diseased oil palms in Krabi, Thailand. Healthy leaves exhibited higher visible and near-infrared radiance compared to diseased ones. Using 113 samples, vegetation indices derived from WorldView-2 imagery achieved 85.98% classification accuracy and a Kappa coefficient of 0.71 (Malinee et al., 2021). BSR caused by *G. boninense* threatens oil palm production, with climate change worsening its impact. Modified oil palms (mOPs) are being developed to resist BSR, although their full deployment will take decades. CLIMEX modeling highlights the significant benefits of mOP in mitigating BSR (Paterson, 2023). A prototype system for early detection of BSR in oil palm trees was developed in Thailand, combining traditional tapping techniques with modern sound analysis. The study utilized machine learning models, including Convolutional Neural Networks (CNN), Support Vector Machine Classifier (SVC), and Multi-Layer Perceptron (MLP). The CNN model achieved the best performance, with 90.73% accuracy in two-class classification (Augsornthip et al., 2024). **Tables 3**, **4** list *Ganoderma*-associated diseases reported in the Lower Mekong Basin, detailing their impact on economically important crops such as oil palm, rubber trees, and black pepper, along with effective prevention and management strategies.

**Table 3:** *Ganoderma*-associated diseases reported in Lower Mekong Basin.

Species	Disease	Host/impact	Key findings	References
*G. applanatum, G. australe, G. colossum*	*Ganoderma* trunk rot, butt and heart rots	Oil palm (*Elaeis guineensis*) forest trees	Major disease in oil palm plantations in Southern Thailand. Causes dangerous forest tree diseases.	Limsrivilai et al., 1980; Watanavanich, 1982; Chalermpongse, 1989, 1999; Klingesorn, 1998; Rodtong et al., 1998
*G. boninense*	Basal Stem Rot (BSR)	Oil palm	Major pathogen causing BSR in oil palm, wilting associated with BSR	Choeyklin et al., 2011; Pornsuriya et al., 2013; Lim et al., 1992
*G. lucidum*	Foot rot, butt rot	Black pepper (*Piper nigrum*), mangrove trees	Causes severe damage to black pepper and butt rot in mangrove species.	Chalermpongse, 1991b
*G. pseudoferreum*	Red root disease	Rubber trees (*Hevea brasiliensis*)	Infects rubber trees through roots, spreading to the trunk, Produces dark red mycelium with light brown edges.	Panchan et al., 1995

**Table 4:** Disease prevention and management of *G. boninense* in Lower Mekong Basin.

Method	Key findings	References
Biological control agents		
- Fungal and actinomycete-based	*Chaetomium* species and *Streptomyces* spp. inhibit *G. boninense*.	Soytong, 2014; Pithakkit et al., 2015
- Potent biocontrol strains	*Streptomyces palmae* CMU-AB204T reduces BSR severity by 75%. Bioactive compounds show strong anti-*Ganoderma* activity.	Sujarit et al., 2020
- *Trichoderma*-based strategies	*T. virens* K1-02 and *T. asperellum* T76-14 suppress *G. boninense* through volatile compounds and enzyme production.	Anothai et al., 2023; Anothai and Chairin, 2024; Samlikamnoed et al., 2023
- Microbial diversity	Asymptomatic trees show higher microbial diversity linked to disease suppression.	Anothai and Chairin, 2022
Chemical and microbial control		
- Plant extracts and fungicides	*Carica papaya* extract shows 41.26% inhibition. Fungicides suppress mycelium growth by up to 98.96%.	Chaisit et al., 2017
- Micronutrient effects	Zn and Mn enhance laccase activity. Micronutrients reduce ligninolytic enzyme activities.	Intara-Anun and Chairin, 2021
Environmental and agronomic factors		
- Soil properties and defenses	Organic matter and nutrients enhance plant defenses.	Anothai and Chairin, 2020
- Temperature and light	Higher temperatures (35°C–40°C)	

## 6 Analysis of bioactive compounds and therapeutic properties of Ganodermataceae by researchers in the Lower Mekong Basin

Herbal medicines commonly used by chronic disease patients vary across countries. In Laos, popular herbs include *Moringa pterygosperma*, *Curcuma longa*, *Centella asiatica*, and *G. lucidum*. In Vietnam, herbs such as *Curcumin*, *Gynostemma pentaphyllum*, *Artichoke*, and *G. lucidum* are prevalent, while in Thailand, frequently used herbs include *Zingiber officinale*, *Andrographis paniculata*, *Curcuma longa*, and *G. lucidum* with 71 herbal products listed in the National List of Essential Drugs. These reflect the region’s widespread reliance on traditional medicine (Peltzer and Pengpid, 2019). Research on the medicinal properties of Ganodermataceae within the Lower Mekong Basin is growing, with studies focusing on its antimicrobial, anti-inflammatory, and anticancer activities, among others. This analysis examines the current body of research conducted in the region, exploring the pharmacological properties of *Ganoderma* and its potential applications in traditional and modern medicine. By synthesizing findings from various studies, the next section provides a comprehensive overview of the medicinal value of the species of *Ganoderma* and allied genera, highlighting their role in the healthcare practices of the Lower Mekong Basin.

### 6.1 Anticancer and tumor suppression potential

*Ganoderma* species, particularly *G. lucidum* have been extensively studied for their medicinal properties. The anticancer and tumor suppression potential of *G. lucidum* is one of its most prominent benefits. Studies have demonstrated that extracts of *G. lucidum* can help extend the lifespan of cancer patients, with some patients living 3–6 months longer and experiencing reduced chemotherapy side effects (Thaithatgom, 1995). Other studies have also observed anticancer activities in *G. lucidum* as part of Thai medicinal teas (Cheeptham and Towers, 2002), while Armassa et al. (2009) found that *G. lucidum* mycelium extracts reduced the viability of breast cancer cells. In addition, research by Soksawatmakhin and Boonyahotra (2013) and Pesee et al. (2013) found that the use of *G. lucidum* extracts resulted in positive therapeutic effects in cancer patients, including improved survival rates and better quality of life. A study found that *Ganoderma lucidum* hot water extract is not mutagenic and shows strong antimutagenic effects in lab tests. It significantly reduced mutagen-induced changes in bacteria and fruit flies, suggesting potential for cancer prevention (Pakdee et al., 2014). *Ganoderma lucidum* was utilized along with other Vietnamese mushrooms to extract 1–3/1–6 β-glucan for encapsulating curcumin into nanoparticles (NanoGluCur) via nano-precipitation. NanoGluCur significantly enhanced curcumin’s water solubility (180-fold) and demonstrated potent anti-cancer effects against Hep-G2 and LU-1 cell lines, with IC50 values of 6.82 and 15.53 mg/ml, respectively. At 40 mg/ml, NanoGluCur reduced tumor size by 59.93% and density by 40.52%, surpassing the performance of free curcumin. These results emphasize *G. lucidum*-derived β-glucan’s potential in improving drug delivery and its applications in functional foods and cancer therapies (Le et al., 2016). During the rainy season, 13 medicinal mushroom specimens were collected in four Thai provinces. *Ganoderma calidophilum* and *Amauroderma rugosum* showed the highest 1,3-β-glucan content, known to inhibit tumor growth by boosting immune responses. Environmental factors such as vegetation, soil, and microclimate contributed to the bioactive compound levels in these mushrooms (Suwanno and Phutphat, 2018).

Two new lanostane triterpenes, 3α,12β,15α-triacetoxy-5α-lanosta-7,9(11),24-trien-26-oic acid (1) and 5α-lanosta-8,24-diene-26,27-dihydroxy-3,7-dione (2), along with sixteen known compounds, were isolated from *G. lucidum*. Compound 1 exhibited significant antitumor activity against PC-3 prostate cancer cells (IC50 = 11.5 μM). Ganoderic acid F (17) demonstrated strong anti-angiogenic effects, inhibiting capillary-like formation in human umbilical vein endothelial cells (Nguyen et al., 2015). A new gymnomitrane-type sesquiterpenoid, gymnomitrane-3α,5α,9β,15-tetrol (1), was isolated from the fruiting body of *G. lucidum*. Its structure was determined using spectroscopic techniques. This compound showed significant inhibition of the growth of epidermal growth factor receptor tyrosine kinase inhibitor (EGFR-TKI)–resistant human lung cancer (A549) and human prostate cancer (PC3) cell lines (Binh et al., 2015). In cancer treatment, *G. lucidum* has shown positive clinical outcomes. Sornprasert and Aroonsrimorakot (2015) demonstrated that the mycelial growth of *G. lucidum* on various substrates can contribute to its clinical efficacy in cancer treatments. Other studies, such as Suprasert et al. (2014, 2015), also confirmed that water extracts and spores of *G. lucidum* could help control gynecological cancers, improving immune function with minimal side effects. In addition, Teekachunhatean et al. (2012) provided insights into the pharmacokinetics of ganoderic acids, suggesting that food intake affects the absorption of these compounds, which could have implications for their clinical effectiveness. Seven novel triterpenoid metabolites, named colossolactones (1−7), were isolated from the fruiting body of *Ganoderma colossum*, from Vietnam, with their structures elucidated using MS and NMR techniques (Kleinwächter et al., 2001). Five compounds, including ergosterol and lanostane derivatives, were isolated from the fruiting body of *G. applanatum* for the first time in Vietnam. Their structures were identified using advanced spectroscopic techniques. Notably, lanosta-7,9(11),24-triene-3,26-diol was reported as a novel compound in this fungus (Van Trung et al., 2018). The anticancer potential of *G. lucidum* triterpenoid extract was evaluated on human Hep-G2 liver cancer cells. The extract demonstrated significant activity, with a half-maximal inhibitory concentration (IC50) value of 67.25 ± 0.82 µg/ml. This suggests that triterpenoids extracted from *G. lucidum* could be considered a promising agent for medicinal treatment, particularly in cancer therapy (Linh et al., 2021). The inhibitory effects of various Thai herbal extracts on the metabolism of anticancer drugs gefitinib, lapatinib, and sorafenib, mediated by the cytochrome P450 enzyme CYP3A, were studied. *Ganoderma lucidum* exhibited minimal impact on the metabolism of these drugs, with IC50 values greater than 10 μg/ml, indicating weak inhibition. In contrast, *Curcuma zedoaria* and *Murdannia loriformis* showed stronger inhibitory effects. The findings suggest potential pharmacokinetic interactions between tyrosine kinase inhibitors and certain herbal extracts, with *G. lucidum* having a lesser effect than the other herbs (Rodseeda et al., 2022). *Ganoderma lucidum* broken spores (GLBS) taken at 750 mg/day for 8 weeks in post-chemotherapy patients increased white blood cell and neutrophil counts, improved quality of life, and caused only mild side effects such as dry mouth. GLBS did not affect liver or kidney function, indicating it may safely support immune recovery (Tangkhaphiphat et al., 2022). Three novel lanostane triterpenoids, ganoellipsic acids A–C, along with seven known *Ganoderma* lanostanoids, were isolated from artificially cultivated *Ganoderma ellipsoideum* (strain BCC 16634). Structural elucidation was conducted using NMR spectroscopy and mass spectrometry, with the absolute configuration of C-25 in compound 1 determined as 25S via the phenylglycine methyl ester (PGME) method (Sappan et al., 2022).

Five steroids, including stigmasterol, ergosterol peroxide, ganodertriol M, lucidumol B, and kansenone, were isolated from *G. australe* fruit bodies in Laos. Structural characterization was performed using HR-MS and NMR spectroscopy. Ergosterol peroxide and kansenone demonstrated notable cytotoxicity against four cancer cell lines (KB, MCF7, SK-LU-1, and Hep-G2). This marks the first report of cytotoxic steroids from *Ganoderma* species in Laos (Keomanykham et al., 2016). *Ganoderma sinense* fruit bodies cultivated in Laos (GS-LW) had the highest triterpenoid content (538.8 µg/g) among samples from different regions. While water-soluble polysaccharide levels were moderate (1.20%), β-glucan levels were comparable (~16%) across all regions. This study highlights the unique bioactive compound profiles in GS-LW, emphasizing its potential for medicinal use (Liu et al., 2017). **Table 5** highlights the anticancer and tumor suppression potential of *Ganoderma* species found in the Lower Mekong Basin.

**Table 5:** Anticancer and tumor suppression potential of *Ganoderma* species found in Lower Mekong Basin.

Species	Bioactive compounds	Findings	Reference
*Amauroderma rugosum*	1,3-β-glucan	Showed the highest 1,3-β-glucan content, known to inhibit tumor growth by boosting immune responses.	Suwanno and Phutphat, 2018
*G. applanatum*	Lanosta-7,9(11),24-triene-3,26-diol, Ergosterol	Five compounds, including novel lanostane derivatives, were isolated and identified using spectroscopic techniques.	Van Trung et al., 2018
*G. australe*	Ergosterol peroxide, Kansenone	Cytotoxicity observed against four cancer cell lines (KB, MCF7, SK-LU-1, Hep-G2).	Keomanykham et al., 2016
*G. calidophilum*	1,3-β-glucan	High β-glucan content contributed to tumor inhibition by boosting immune responses.	Suwanno and Phutphat, 2018
*G. colossum*	Colossolactones (1–7)	Seven novel triterpenoids were isolated and structurally elucidated.	Kleinwächter et al., 2001
*G. ellipsoideum*	Ganoellipsic acids A–C	Three novel lanostane triterpenoids were isolated, with structures determined using NMR and mass spectrometry.	Sappan et al., 2022
*G. lucidum*	Triterpenoids, Ganoderic acid F, Gymnomitrane-type sesquiterpenoid	Exhibited anticancer activity, reduced chemotherapy side effects, and increased survival in cancer patients.	Multiple references below
	1-3/1-6 β-glucan, Curcumin	NanoGluCur enhanced curcumin solubility and showed potent anticancer effects.	Le et al., 2016
	Ganoderic acid F	Strong anti-angiogenic effects; inhibited capillary formation in endothelial cells.	Nguyen et al., 2015
	Gymnomitrane-3α,5α,9β,15-tetrol	Inhibited EGFR-TKI-resistant lung and prostate cancer cell growth.	Binh et al., 2015
	Triterpenoid extract	Significant anticancer activity against Hep-G2 liver cancer cells (IC50 = 67.25 ± 0.82 µg/ml).	Linh et al., 2021
	Various compounds	Minimal impact on anticancer drug metabolism (IC50 > 10 μg/ml).	Rodseeda et al., 2022
	Broken spores (GLBS)	Increased WBC and neutrophil counts in post-chemotherapy patients, improving immune recovery.	Tangkhaphiphat et al., 2022
*G. sinense*	Triterpenoids, Polysaccharides, β-glucan	Highest triterpenoid content in Laos samples, with strong bioactive potential.	Liu et al., 2017

### 6.2 Immunomodulatory effects

Beyond cancer, *G. lucidum* is known for its immunomodulatory properties. Studies like Mizuno et al. (1997) showed that crude *G. lucidum* extracts can restore immune function in immunosuppressed individuals, enhancing T-cell activity. Further research by Futrakul et al. (2003) highlighted the ability of *G. lucidum* to modulate immunocirculatory balance, demonstrating its potential in treating nephrotic syndrome and other immune-related conditions. Sornprasert and Aroonsrimorakot (2015) also observed significant immunomodulatory effects when *G. lucidum* was cultivated in certain substrates, emphasizing its clinical potential. Chronic fatigue syndrome (CFS) patients in a study received either *Ganoderma lucidum* extract or a placebo. After 4 weeks, the *G. lucidum* group showed significantly improved quality of life (*p* = 0.005) and reduced fatigue (*p* = 0.010) compared to the placebo. After 12 weeks, serum cortisol levels rose in the *G. lucidum* group, with higher satisfaction reported by these participants (*p* < 0.001). Side effects (diarrhea and nausea) were similar in both groups. Findings suggest that *G. lucidum* extract may be effective in alleviating fatigue and enhancing the quality of life for CFS patients (Soksawatmakhin and Boonyahotra, 2013).

### 6.3 Neuroprotective and cognitive benefits

The neuroprotective effects of *G. lucidum* have also been a subject of interest, with studies such as Porntip et al. (2006) showing that *G. lucidum* extracts promote neuroprotection in neuronal cultures, suggesting benefits in cognitive health. Pinhewa et al. (2008) found that *G. lucidum* extracts increased the expression of amyloid precursor protein (APP) and promoted sAPPα secretion, both of which are linked to improved cognitive function. In addition, Boonyanuphap and Hansawasdi (2011) showed that *G. lucidum* contains β-glucans, which are known to support cognitive health, potentially aiding in memory enhancement.

Neural stem cells (NSCs) are promising for treating neurological disorders due to their self-renewal and pluripotency, but exogenous sources are often needed for effective therapy. This study demonstrated that *G. lucidum* extract at 500 μg/ml significantly enhanced NSC proliferation, with isolated cells forming neurospheres, expressing neural markers, and differentiating into GFAP-positive cells (Dan et al., 2017). *Ganoderma lucidum* extracts, at doses of 200–400 mg/kg, effectively reduced morphine addiction and improved morphine-induced memory impairments in animal models. Using the conditioned place preference model and memory tests (Y maze, novel recognition, and Morris water maze), the extracts demonstrated the ability to prevent addiction and enhance short-term, visual, and long-term memory. These findings suggest *G. lucidum* extracts as a potential natural treatment for drug addiction and memory loss (Tran et al., 2021).

### 6.4 Antioxidant and anti-inflammatory properties

#### 6.4.1 Amauroderma subresinosum

*Amauroderma subresinosum (= Ganoderma subresinosum)* polysaccharides were extracted and characterized, revealing glucose-rich fractions with strong antioxidant activity. The primary fractions, ASF-1, ASF-3, and ASF-7, demonstrated significant DPPH and ABTS radical scavenging, suggesting their potential as antioxidant-rich functional food ingredients (Nguyen et al., 2023f).

#### 6.4.2 Ganoderma australe

A new lanostane triterpene and three known compounds were isolated from cultivated fruiting bodies of *G. australe*. NMR and mass spectrometry confirmed their identities, with a revised olefinic geometry of methyl australate from 20(22)Z to 20(22) E. These compounds differ from lanostanes previously found in mycelial cultures of the same strain. Given their known antioxidant and anti-inflammatory properties, these newly identified compounds may contribute to these bioactivities, although further studies are needed (Isaka et al., 2018). An HPLC-DAD method was developed for quality control of *G. lucidum* (and related species), focusing on 14 triterpene compounds. The method showed good linearity, low detection limits, and recovery rates between 97.09% and 100.79%. Significant differences in triterpene content were found, with wild *G. lucidum* having higher levels than cultivated samples. *G. australe* had four times the triterpene content of wild *G. lucidum* (Ha et al., 2015).

#### 6.4.3 Ganoderma lucidum

In terms of antioxidant and anti-inflammatory properties, *G. lucidum* is a powerful agent. Studies by Armassa et al. (2009) revealed that extracts from *G. lucidum* exhibited significant antioxidant activity, neutralizing free radicals and showing promise in reducing oxidative stress. Other studies, such as Sa-ard et al. (2014), confirmed that the antioxidant potential of *G. lucidum* could play a role in managing oxidative stress-related conditions. Dalodom et al. (2010) found that hot water extracts of *G. lucidum* exhibited both antioxidant properties and a lack of mutagenic effects, making them suitable for therapeutic use. Thai *G.lucidum* (G2), cultivated as part of Thailand’s Royal Project since 1988, was evaluated for safety and efficacy. Comparing the fruiting body and mycelium extracts, both showed no mutagenic effects, notable antioxidant activity, and mild iron-chelating properties, with the fruiting body having superior antioxidant capacity. Non-mutagenic doses also displayed cytotoxicity to lung carcinoma cells, providing useful insights for developing safe health-promoting products (Dalodom et al., 2010). The polysaccharide content, antioxidant activity, and cytotoxicity of *G. lucidum* mycelium extracts were investigated. The water extract exhibited a higher polysaccharide content and stronger antioxidant activity compared to the ethanol extract. In addition, it demonstrated significant cytotoxic effects on HeLa cervical cancer cells, reducing cell viability by 56.30% at 1 mg/ml, whereas the ethanol extract showed cytotoxicity to normal cells. These findings suggest that *G. lucidum* mycelium extracts possess potential antioxidant and anticancer properties, making them promising candidates for pharmaceutical and functional food applications (Sanmanoch et al., 2024). Crude proteins from *G. lucidum* mycelia and fruiting bodies showed strong antioxidant and antibacterial activities. The mycelia protein had better antioxidant effects with IC50 values of 2.47 μg/ml (ABTS•+) and 2.5 μg/ml (DPPH•), compared to the fruiting body protein. Both proteins exhibited antibacterial activity, and the mycelia protein also protected DNA from hydroxyl radicals. Partial purification revealed a major protein of 45 kDa. These results suggest that *G. lucidum* protein extracts have potential as antioxidant and antibacterial agents (Sa-Ard et al., 2015). *Ganoderma lucidum*, a functional food ingredient, contains hydrolysates with diverse biological activities. This study identified and modified a peptide (VDLPTCKGF), synthesizing seven variants. Among these, three exhibited antioxidant activity, with VDLPTC showing the strongest capacity and intracellular ROS suppression, highlighting its potential for developing novel functional food products (Krobthong and Yingchutrakul, 2020). An HPLC-DAD method was developed for quality control of *G. lucidum* (and related species), focusing on 14 triterpene compounds. The method showed good linearity, low detection limits, and recovery rates between 97.09% and 100.79%. Significant differences in triterpene content were found, with wild *G. lucidum* having higher levels than cultivated samples. *G. australe* had four times the triterpene content of wild *G. lucidum* (Ha et al., 2015). An extract from *G. lucidum and Cordyceps militaris* was developed and analyzed for its composition, antioxidant activities, and protective effects. Using LC-QTOF MS, 94 compounds were identified, including ferulic acid, cinnamic acid, ganoderic acid A, adenosine, and cordycepin. The extract demonstrated antioxidant properties, scavenged free radicals, reduced ferric ions, and protected human fibroblasts from oxidative stress, reducing cell death by 21%–22%. These results highlight its potential applications in functional foods and pharmaceuticals (Nguyen et al., 2024a). Methanol extracts of *G. mastoporum* fruiting bodies from Vietnam yielded eight compounds, including three triterpenoids and five steroids. Among these, ergosta-4,6,8(14),22-tetraen-3-one showed the strongest inhibitory effects on superoxide anion generation and elastase release, with IC50 values of 2.30 ± 0.38 and 1.94 ± 0.50 µg/ml, respectively (Thang et al., 2013).

#### 6.4.4 *Ganoderma neo-japonicum*, *G. pfeifferi* and *G. tropicum*

Hot-water extracts of *G. neo-japonicum* (GnJ) and *G. lucidum* (GL) were examined for their functional composition and antioxidant properties. While both species showed antioxidant activity, GnJ exhibited a FRAP value of 181.1. The maximum DPPH and ABTS scavenging rates for GnJ were 27.9% and 76.0%, respectively, at 5 mg/ml concentrations. These values were lower compared to the antioxidant activity observed in *G. lucidum* (Ayimbila et al., 2023). Three triterpenoids and three steroids were identified from the fruiting bodies of *G. pfeifferi* collected in Vietnam, and their effects on nitric oxide (NO) production were evaluated. The findings suggest that certain compounds from this fungus could serve as potential leads for the development of new anti-inflammatory drugs (Kuo et al., 2016). A new compound, 3β-acetoxylanosta-7,9(11),24-triene-26-al, along with seven known compounds, was isolated from *G. tropicum* in Tay Nguyen, Vietnam. Structural identification was achieved using NMR and mass spectrometry. Compounds 2–4 and 6–8 showed dose-dependent enhancement of nitroblue tetrazolium (NBT) reduction in yeast-stimulated RAW 246.7 cells (Nguyen et al., 2020). **Table 6** explores the antioxidant and anti-inflammatory properties of *Ganoderma* species found in the Lower Mekong Basin, emphasizing their bioactive compounds and potential therapeutic applications.

**Table 6:** Antioxidant and anti-inflammatory properties of *Ganoderma* species found in Lower Mekong basin.

Species	Bioactive compounds	Key findings	References
*Amauroderma subresinosum*	Polysaccharides (ASF-1, ASF-3, ASF-7)	Strong antioxidant activity; effective DPPH and ABTS radical scavenging.	Nguyen et al., 2023f
*G. australe*	Lanostane triterpenes, methyl australate	Isolated a new lanostane triterpene; *G. australe* had four times the triterpene content of wild *G. lucidum*.	Isaka et al., 2018; Ha et al., 2015
*G. lucidum*	Polysaccharides, triterpenes, peptides (VDLPTCKGF), proteins	Exhibits antioxidant, anti-inflammatory, anticancer, and antibacterial properties.	Armassa et al. (2009); Dalodom et al., 2010; Sanmanoch et al., 2024; Sa-Ard et al., 2015; Krobthong and Yingchutrakul, 2020
*G. mastoporum*	Triterpenoids, steroids (ergosta-4,6,8(14),22-tetraen-3-one)	Extracts inhibited superoxide anion generation and elastase release.	Thang et al., 2013
*G. neo-japonicum*	Polysaccharides, triterpenes	Showed antioxidant activity with FRAP value of 181.1.	Ayimbila et al., 2023
*G. pfeifferi*	Triterpenoids, steroids	Identified bioactive compounds with anti-inflammatory potential.	Kuo et al., 2016
*G. tropicum*	3β-acetoxylanosta-7,9(11),24-triene-26-al, triterpenoids	New compounds enhanced nitroblue tetrazolium (NBT) reduction in immune cells.	Nguyen et al., 2020

### 6.5 Antimicrobial effects

#### 6.5.1 Antiviral activity

Four new lanostane triterpenes (colossolactones V–VIII) and a known compound (colossolactone E) were isolated from *G. colossum*. Their structures and absolute configurations were identified using spectroscopic techniques. Along with two previously isolated compounds, these were evaluated for HIV-1 protease inhibition, with the most active showing IC50 values of 5–13 µg/ml (El Dine et al., 2008). Ganomycin I and ganomycin B, isolated from *G. colossum*, inhibited HIV-1 protease with IC50 values of 7.5 and 1.0 μg/ml, respectively. Kinetic studies showed that ganomycin B competitively inhibited the enzyme’s active site, while schisanlactone A, another compound from the same fungus, acted as a dimerization inhibitor (IC50 = 5.0 μg/ml). Virtual docking confirmed these inhibitory mechanisms (El Dine et al., 2009).

#### 6.5.2 Antibacterial activity

Cheeptham and Towers (2002) showed that *G. lucidum* was effective against *Staphylococcus aureus*, a common bacterial pathogen, in Thai medicinal teas. *G. australe* extracts inhibited *Micrococcus luteus*, *Bacillus subtilis*, *S. aureus*, and *Salmonella* ser. *typhimurium* but were ineffective against *E. coli* and *Pseudomonas aeruginosa*. This is the first report of its antimicrobial activity (Luangharn et al., 2017). *Ganoderma neojaponicum* demonstrated higher minimum inhibitory concentrations (MICs) ranging from 2.5 to 5 mg/ml against pathogens, compared to *G. lucidum*. Scanning electron microscope (SEM) analysis further revealed that *G. neojaponicum* caused cell lysis and wall shrinkage in the pathogens, underscoring its potential antibacterial properties, although it was less potent than *G. lucidum* (Ayimbila et al., 2023). The methanol extract of *G. lucidum* exhibited significant antibacterial activity against several foodborne pathogens, including both Gram-positive and Gram-negative bacteria, suggesting its potential as a natural antibacterial agent (Chaiharn et al., 2018). *Ganoderma* metabolites showed strong antibacterial potential against *S. aureus*, with ganosinoside A exhibiting the highest affinity for clumping factor A. In addition, *G.lingzhi* and *A. subresinosum* strains from Vietnam demonstrated antibacterial activity, suggesting their value as sources for developing antibiotics (Nguyen et al., 2024b). Four new 3,4-seco-27-norlanostane triterpenoids (ganoboninketals E and F, ganoboninones G and H) and two known derivatives, along with a new C30 lanostane and twelve known lanostanes, were isolated from *G. orbiforme* fruiting bodies. Furthermore, three known meroterpenoids (fornicin A, ganomycin B, and ganomycin I) were identified. The structures of the compounds were determined using NMR, mass spectrometry, and chemical correlations. Biological testing revealed that ganomycin I exhibited moderate activity against Gram-positive bacteria (Li et al., 2018).

#### 6.5.3 Antimalarial activity

A revised structure of colossolactone G and seven new triterpene lactones, ganodermalactones A−G, and five known triterpene lactones and ergosterol were isolated from cultured *Ganoderma* sp. KM01. Structures were identified using spectroscopic methods, with x-ray analysis confirming configurations for compounds 3, 7, and 8. Compounds 7, 10, and 12 showed antimalarial activity against *Plasmodium falciparum* with IC50 values between 6.0−10.0 μM (Lakornwong et al., 2014). Three new lanostane triterpenoids—ganopyrone A, ganocolossusin I, and ganodermalactone Y—were isolated from the cultivated fruiting bodies of *Ganoderma colossus* TBRC-BCC 17711. Ganopyrone A has a unique polycyclic structure with an α-pyrone ring and a C-18/C-23 bond. It demonstrated antimalarial activity against the multidrug-resistant *P. falciparum* K1 strain (IC50 7.8 μM) with low cytotoxicity on Vero cells (IC50 103 μM) (Chanphen et al., 2024). From the cultivated fruiting bodies of *Ganoderma weberianum*, two lanostane dimers, ganoweberianones A and B, along with seven new lanostanes (ganoweberianic acids A–G) and three known compounds, were isolated. Ganoweberianone A showed notable antimalarial activity against the multidrug-resistant strain *P. falciparum* K1 (IC50 = 0.050 μM). A semisynthesis method for ganoweberianone A was also developed using acid-catalyzed transesterification (Isaka et al., 2020a). Sixteen new lanostane-type triterpenoids (1–16) and fourteen known compounds were isolated from the cultivated fruiting bodies of *Ganoderma casuarinicola*. The structures of these compounds were determined using NMR spectroscopy and mass spectrometry. Two of the compounds, 9 and 10, exhibited antimalarial activity with IC50 values of 9.7 and 9.2 μg/ml, respectively (Isaka et al., 2020b). Eight new highly modified lanostane triterpenoids, ganoboninketals G–K (1–5), ganoboninone I (6), ganoderlactone G (7), and (24E)-3,11-dioxolanosta-8,24-dien-26-oic acid (8), were isolated from the fruiting bodies of *Ganoderma* cf. *hochiminhense*. Their structures were determined using NMR spectroscopy and mass spectrometry. Ganoboninketals G (1), H (2), and J (4) showed antimalarial activity against the multidrug-resistant *P. falciparum* K1 strain, with IC50 values of 17, 16, and 5.1 μM, respectively (Palasarn et al., 2024). Ten new lanostane-type triterpenoids (1–10) and 15 known lanostanes were isolated from cultivated *Ganoderma* sp. BCC 21329. Their structures were determined using NMR, mass spectrometry, and Mosher’s method. Compounds 1, 3, 5, and 7 exhibited moderate antimalarial activity with IC50 values of 3.8–7.6 μg/ml (Isaka et al., 2020c). Eleven novel lanostane triterpenoids, including a unique chlorinated derivative, were isolated from *Ganoderma mbrekobenum*. Structural elucidation was performed using NMR, mass spectrometry, and ECD calculations, with chemical derivatization confirming the C-20 configuration in the most abundant compound. Two of the compounds showed moderate antimalarial activity (Yangchum et al., 2022). Eight new lanostane triterpenoids were isolated from *G. weberianum* TBRC-BCC 60642 cultures, with structural differences observed between mycelial cultures and fruiting bodies. Compounds 2, 3, and 6 exhibited moderate antimalarial activity against *P. falciparum* K1, with IC_50_ values of 10–15 μM (Chinthanom et al., 2022a). Colossolactone J, a newly identified lanostane-type triterpenoid, was purified from the fruiting body of Ganoderma colossus via silica gel column chromatography and preparative HPLC. Its molecular structure and absolute configuration were determined using spectroscopic techniques, including NMR and the modified Mosher’s method (Chinthanom et al., 2022b).

#### 6.5.4 Antitubercular activity

Lanostane triterpenoids with strong anti-tuberculosis (anti-TB) activity were isolated from the mycelial cultures of *G. australe* strain TBRC-BCC 22314. To evaluate its use in anti-TB products, a chemical analysis of autoclaved and non-autoclaved mycelial powders was conducted. Both powders showed the same anti-TB activity (MIC 3.13 μg/ml) against *Mycobacterium tuberculosis* H37Ra. However, sterilization led to unique chemical conversions of lanostanes. The main active lanostane, ganodermic acid S, was also effective against extensively drug-resistant (XDR) strains of *M. tuberculosis* (Chinthanom et al., 2023a). Antitubercular research on lanostane triterpenoids from *Ganoderma* submerged cultures examined three strains: *G. orbiforme* BCC 22325, *Ganoderma* sp. BCC 60695, and *G. australe* BCC 22314. Fourteen new lanostane triterpenoids and 35 known compounds were isolated and tested against *M. tuberculosis* H37Ra. Structure–activity relationship analysis identified 3β- and 15α-acetoxy groups as essential for antimycobacterial activity, with the most potent compound being (24E)-3β,15α-diacetoxylanosta-7,9(11),24-trien-26-oic acid (Isaka et al., 2017a). Sixteen new lanostane triterpenoids (1–16) and 26 known compounds (17–42) were isolated from *Ganoderma* sp. BCC 16642. The antitubercular activities of these compounds were tested against *M. tuberculosis* H37Ra, and structure–activity relationships were proposed based on the results (Isaka et al., 2016). *Ganoderma weberianum* yielded 11 novel lanostane dimers (ganoweberianones C-H and isoganoweberianones A/B/D/G/H), 6 new ganodermanontriol derivatives, and 5 ganoweberianic acids. A semisynthetic condensation method aided structural characterization. Notably, ganoweberianone D (IC50 0.057 μM) and isoganoweberianone D (IC50 0.035 μM) exhibited potent antiplasmodial activity against multidrug-resistant *P. falciparum* K1 with minimal cytotoxicity (Vero cell IC50 8.1-19 μM), highlighting their therapeutic potential (Chinthanom et al., 2023b). Seven new lanostane triterpenoids (1–7) were isolated from cultivated *Ganoderma wiiroense* (strain TBRC-BCC 60613) and identified using NMR and mass spectrometry. The absolute configuration of C-23 in compound 1 was determined as 23S. Among the isolates, compound 7 exhibited antitubercular activity against *M. tuberculosis* H37Ra, with an MIC of 50 μg/ml (Yangchum et al., 2023). Antitubercular lanostane triterpenoids were isolated from the mycelial cultures of *G. australe* and structurally modified through semisynthesis. One of the synthetic compounds, GA003 (9), demonstrated greater potency against *M. tuberculosis* H37Ra than the natural lead compound (1). GA003 also exhibited significant activity against the virulent H37Rv strain and extensively drug-resistant tuberculosis strains (Chinthanom et al., 2021a). Three new lanostane triterpenoids, along with 21 known compounds, were isolated from the fruiting bodies of *Ganoderma sichuanense*. The absolute configuration at C-25 of ganoderic acid A and its derivatives was determined to be 25R using the phenylglycine methyl ester (PGME) method. Among the isolated compounds, ganoderiol F demonstrated the strongest activity against *M. tuberculosis* H37Ra, with a MIC value of 0.781 μg/ml (Chinthanom et al., 2021b). A new 3,4-seco-27-norlanostane triterpene, ganoboninketal D (1), and a new lanostane, (24S)-3-oxo-7α,24,25-trihydroxylanosta-8-ene (2), along with six known lanostanes, were isolated from *G. orbiforme* fruiting bodies. Structures were determined using NMR and mass spectrometry, with compounds 1 and 2 confirmed through chemical comparisons. Only compound 8 showed weak antitubercular activity, while the others were inactive against *M. tuberculosis* and *P. falciparum* (Isaka et al., 2017b).

#### 6.5.5 Antiprotozoal activity

*Ganoderma lucidum* extracts showed potent antiprotozoal activity against *Blastocystis hominis* subtype 3, with a minimum inhibitory concentration of 62.5 μg/ml. At higher concentrations, the extract inhibited parasite growth by up to 90% within 12h. The key compound identified in *G. lucidum* was versalide, highlighting its potential for medicinal applications (Kaewjai et al., 2023). **Table 7** examines the antimicrobial properties of *Ganoderma* species found in the lower Mekong region, highlighting their bioactive compounds and effectiveness against pathogenic microbes.

**Table 7:** Antimicrobial properties of *Ganoderma* species found in lower Mekong region.

Category	Species/strain	Key findings/compounds	Bioactive compounds	References
Antibacterial activity	*G. lucidum*	Effective against *S. aureus* in Thai medicinal teas.	Unknown	Cheeptham and Towers (2002)
		AgNPs synthesized using ultrasonic-assisted heating reflux showed strong antibacterial and antifungal properties.	Silver nanoparticles	Nguyen et al. (2023h)
	*G. australe*	Extracts inhibited *M. luteus*, *B. subtilis*, *S. aureus*, and *S. typhimurium*. Ineffective against *E. coli* and *P. aeruginosa*.	Unknown	Luangharn et al. (2017)
	*G. neojaponicum*	Higher MICs (2.5–5 mg/ml) against pathogens. SEM analysis revealed cell lysis and wall shrinkage.	Unknown	Ayimbila et al. (2023)
	*G. lucidum*	Methanol extract showed antibacterial activity against foodborne pathogens. Highest antioxidant potential (IC50: 2.81–10.57 mg/ml).	Unknown	Chaiharn et al. (2018)
	*G. lingzhi*	Metabolites showed strong antibacterial activity against *S. aureus*	Ganosinoside A	Nguyen et al. (2024)
Antiviral activity	*G. colossum*	Four new lanostane triterpenes showed HIV-1 protease inhibition (IC50: 5–13 µg/ml).	Colossolactones V–VIII, Colossolactone E	El Dine et al. (2008)
		Ganomycin I and ganomycin B inhibited HIV-1 protease (IC50: 7.5 and 1.0 µg/ml). Schisanlactone A acted as a dimerization inhibitor (IC50: 5.0 µg/ml).	Ganomycin I, Ganomycin B, Schisanlactone A	El Dine et al. (2009)
Antimalarial activity	*Ganoderma* sp. KM01	Seven new triterpene lactones and five known compounds showed antimalarial activity (IC50: 6.0–10.0 µM).	Ganodermalactones A–G	Lakornwong et al. (2014)
	*G. colossus* TBRC-BCC 17711	Three new lanostane triterpenoids showed antimalarial activity (IC50: 7.8 µM).	Ganopyrone A, Ganocolossusin I, Ganodermalactone Y	Chanphen et al. (2024)
	*G. casuarinicola*	Sixteen new lanostane triterpenoids showed antimalarial activity (IC50: 9.7 and 9.2 µg/ml).	lanostane triterpenoids	Isaka et al. (2020a)
	*G.* cf. *hochiminhense*	Eight new lanostane triterpenoids showed antimalarial activity.	Ganoboninketals G–K, Ganoboninone I, Ganoderlactone G	Palasarn et al. (2024)
Antitubercular activity	*G. australe*	Lanostane triterpenoids showed anti-TB activity (MIC: 3.13 µg/ml). Ganodermic acid S was effective against XDR strains.	Ganodermic acid S	Chinthanom et al. (2023a)
	*G. orbiforme*	Fourteen new lanostane triterpenoids and 35 known compounds. (24E)-3β,15α-diacetoxylanosta-7,9(11),24-trien-26-oic acid was the most potent.	24E-3β,15α-diacetoxylanosta-7,9(11),24-trien-26-oic acid	Isaka et al. (2017)
	*G. sichuanense*	Three new lanostane triterpenoids and 21 known compounds. Ganoderiol F showed strong anti-TB activity (MIC: 0.781 µg/ml).	Ganoderiol F	Chinthanom et al. (2021a)
Antiprotozoal activity		Extracts showed antiprotozoal activity against *B. hominis* subtype 3 (MIC: 62.5 µg/ml)	Versalide	Kaewjai et al. (2023)

### 6.6 Metabolic and physiological regulation

Researchers cultivated mycelia from a wild Thai mushroom, *G. australe*, identified through PCR and morphological analysis. LC-MS/MS revealed bioactive compounds, including lovastatin. The extracts inhibited HMG-CoA reductase and increased HDL production in HepG2 spheroids to 71.35%, compared to 33.26% in controls and 32.13% with lovastatin alone. This study highlights the potential of *G. australe* as a functional food for hypercholesterolemia prevention (Wongkhieo et al., 2023).

The metabolic and physiological benefits of *G. lucidum* are also significant. Sornprasert (1995) highlighted its high protein content, which adds to its nutritional value. Studies like Futrakul et al. (2003) demonstrated that *G. lucidum* could suppress proteinuria and improve vascular function in patients with nephrotic syndrome, underscoring its potential for managing metabolic conditions. In patients with nephrotic syndrome and focal segmental glomerulosclerosis (FSGS) who had persistent proteinuria despite treatment with prednisolone, cyclophosphamide, and vasodilators, *G. lucidum* was introduced as an additional therapy. Initially, these patients showed increased endothelial cell cytotoxicity and an imbalance in cytokines, with elevated TNF-alpha and low IL-10 levels. Treatment with *G. lucidum* reduced endothelial cytotoxicity, restored cytokine balance, and successfully decreased proteinuria in all 14 patients (Futrakul et al., 2004). Ganomycin I (GMI) from *G. lucidum* inhibits RANKL-induced osteoclast formation, bone resorption, and related signaling pathways (MAPKs, c-Fos, NFATc1) without affecting cell viability. It downregulates osteoclast-specific genes, suggesting its potential as an anti-osteoporotic agent (Tran et al., 2019). *Ganoderma lucidum* was studied as a natural hypolipidemic agent for reducing lipid accumulation. Optimized enzyme and time conditions produced hydrolysates with high yield and hydrolysis, encapsulated in nanoscale liposomes. These liposomes promoted triglyceride breakdown in adipocytes and reduced lipid levels without harming cell viability. Proteomic analysis highlighted key proteins affected by treatment, suggesting the potential for *G. lucidum* hydrolysates in obesity management (Krobthong et al., 2021). The anti-diabetic potential of *G. lucidum* methanol-extracted components (1–15) were evaluated using molecular docking simulations. Component 1 (Butyl lucidenate P) showed the best α-glucosidase inhibition (DS -12.8 kcal/mol, RMSD 1.23 Å). QSARIS and ADMET analyses confirmed their biocompatibility and pharmacological suitability. Quantum-based analysis further supported the potential of components 1, 2, 11, and 13 for anti-diabetic applications (Nguyen et al., 2023g). Wild *Ganoderma* strains from northern Thailand, including *G. orbiforme* and *G. sichuanense* were rich in fiber, protein, fat, and carbohydrates. Both showed strong alpha-glucosidase inhibition, outperforming acarbose (Wannasawang et al., 2023).

Natural PPAR ligands were investigated in Vietnamese medicinal plants, fungi, and foods as potential alternatives to synthetic ligands for obesity and metabolic syndrome prevention. The extracts exhibited varying levels of PPAR agonistic activity, with fungi and certain plants showing strong PPARδ activity, indicating their potential as natural resources for preventing metabolic disorders (Ngoc et al., 2019). In Cambodia, a study on TCAM (Traditional, Complementary, and Alternative Medicine) used among patients with chronic diseases found that 27% consulted TCAM providers in the past year, with herbalists (17.3%) being the most common. The use of herbal medicine was reported by 41%, vitamins by 26.5%, and other supplements by 9.7%. The study also highlighted self-help practices such as praying for health (30.1%) and meditation (13.9%). Factors associated with TCAM use in Cambodia included older age, rural residence, and higher formal education. In addition, having two or more chronic conditions was linked to higher use of TCAM providers and products (Peltzer et al., 2016). The prevalence of herbal and dietary supplement (HDS) use among Thai patients with type 2 diabetes mellitus (DM) was found to be 61%, with 28% actively consuming them. Many patients did not inform their physicians, often citing a lack of concern, and 73% were unaware of potential drug-herb interactions. Common HDS included drumstick tree, turmeric, bitter gourd, and *Ganoderma*, influenced by social media and peer recommendations. These findings emphasize the importance of addressing HDS use in clinical practice to improve safety and glycemic management for Thai DM patients (Prasopthum et al., 2022). **Table 8** explores the metabolic and physiological benefits of *Ganoderma* species found in the Lower Mekong Basin.

**Table 8:** Metabolic and physiological benefits of *Ganoderma* species found in the Lower Mekong basin and their bioactive compounds.

Species	Metabolic and physiological regulation	Reference
*Ganoderma australe*	Mycelial extract contained bioactive compounds, including lovastatin. Inhibited HMG-CoA reductase and increased HDL production in HepG2 spheroids, suggesting potential for hypercholesterolemia prevention.	Wongkhieo et al., 2023
*G. lucidum*	High protein content enhances nutritional value.	Sornprasert, 1995
	Suppressed proteinuria and improved vascular function in nephrotic syndrome patients.	Futrakul et al., 2003
	Reduced endothelial cytotoxicity and restored cytokine balance in patients with nephrotic syndrome and focal segmental glomerulosclerosis (FSGS).	Futrakul et al., 2004
	Methanol-extracted components (1–15) were evaluated for anti-diabetic potential. Butyl lucidenate P showed strong α-glucosidase inhibition. QSARIS, ADMET, and quantum analysis confirmed pharmacological suitability.	Nguyen et al., 2023g
	Strains used in Cambodia as part of TCAM for chronic disease management.	Peltzer et al., 2016
	Commonly used as a herbal dietary supplement by type 2 diabetes patients, but many users were unaware of potential drug-herb interactions.	Prasopthum et al., 2012
*G. orbiforme*	Showed strong alpha-glucosidase inhibition, outperforming acarbose, indicating potential for blood sugar regulation.	Wannasawang et al., 2023
*G. sichuanense*	Rich in fiber, protein, fat, and carbohydrates; demonstrated strong alpha-glucosidase inhibition.	Wannasawang et al., 2023
*G. subresinosum*	A natural hypolipidemic agent; enzymatically hydrolyzed extracts encapsulated in nanoscale liposomes promoted triglyceride breakdown in adipocytes, reducing lipid accumulation.	Krobthong et al., 2021
*Ganoderma* sp	Natural PPAR ligands in Vietnamese medicinal fungi and plants showed strong PPARδ activity, indicating potential for metabolic disorder prevention.	Ngoc et al., 2019

### 6.7 Toxicity and side effects

While *G. lucidum* offers numerous health benefits; its toxicity and side effects should not be overlooked. Wanachiwanawin et al. (2006) reported a case of pseudoparasitosis linked to the consumption of *G. lucidum* spores, while Wanmuang et al. (2007) noted hepatotoxicity in patients consuming *G. lucidum* powder. These studies indicate that, while generally safe, *G. lucidum* should be used with caution in individuals with liver problems or those who are particularly sensitive to its effects. A 49-year-old man in Thailand with non-Hodgkin’s lymphoma developed chronic diarrhea, which was linked to his consumption of powdered *G. lucidum* extract as a dietary supplement. Stool tests showed numerous *G. lucidum* spores, initially mistaken for parasitic infections. His diarrhea improved when he stopped ingesting the mushroom spores, and no further *Ganoderma* spores were found. This case highlights the importance of distinguishing fungal spores from parasitic organisms in stool samples to avoid misdiagnosis (Wanachiwanawin et al., 2006). Heavy metal levels and toxicity on *G. lucidum* were evaluated, showing trace amounts in fruit bodies and substrates. Toxicity to mycelial growth ranked highest for Hg and Cd, with Zn uptake reaching over 60% from substrates, resulting in high accumulation in fruitbodies and basidiospores (Tham et al., 1999). Hepatotoxicity linked to *G. lucidum* powder was first reported in 2004 with a Hong Kong (China) patient, followed by a fatal case of fulminant hepatitis in 2005. Both patients had previously consumed traditionally boiled *G. lucidum* without adverse effects. However, after switching to powdered *G. lucidum* for 1–2 months, they developed hepatotoxicity. The potential risks of *G. lucidum* powder, particularly when combined with other medications, warrant careful monitoring in the future (Wanmuang et al., 2007). Hepatoprotective effects of *G. lucidum* from dead ironwood trees in Vietnam’s Central Highlands were evaluated in mice with cyclophosphamide-induced liver toxicity (150 mg/kg, intraperitoneal). Oral administration of the extract (120, 230, and 330 mg/kg body weight) significantly reduced liver malondialdehyde (MDA) levels and restored glutathione (GSH) levels, showing efficacy comparable to silymarin. Histopathological analysis confirmed these findings, suggesting *G. lucidum* as a potential natural liver-protective agent (Pham et al., 2016). Wall-broken *G. lucidum* spores were processed via autoclaving and extracted with ethanol at varying concentrations. The 70% ethanol extract showed the highest triterpenoid content, predominantly ganoderic acid A, and the strongest antioxidant activity in the DPPH assay. Safe in mice at a dose of 2,000 mg/kg, it also demonstrated hepatoprotective effects by preventing serum ALT and AST elevation and reducing oxidative stress markers in cyclophosphamide-induced liver injury (Thuy et al., 2022).

### 6.8 Nutritional and health benefits

Nutritionally, *G. lucidum* is an excellent source of health-promoting compounds. Armassa et al. (2005) recognized it as a valuable nutritional food and alternative medicine for promoting longevity and overall well-being. Boonyanuphap and Hansawasdi (2011) highlighted the presence of β-glucans in *G. lucidum*, supporting its use as a supplement for improving health. Pinitsoontorn et al. (2012) also found that *G. lucidum* extracts could help prevent the formation of calcium oxalate stones, making it a beneficial supplement for kidney health. Wannasupchue et al. (2011) demonstrated that adding crushed *G. lucidum* to smoked fish sausages increased nutritional value and helped retard lipid oxidation, showcasing its role in food preservation. The antler-type fruiting body of *G. lucidum* was used to extract β-glucan (BG) for cosmeceutical applications. The extract, containing 40.57% BG and 7.47% protein, demonstrated anti-tyrosinase and antioxidation activities, suggesting potential for skin whitening. It also showed moderate anti-collagenase, anti-elastase, and anti-hyaluronidase effects. Skin irritation tests were negative, and BG had no significant effect on cell viability. The extract outperformed commercially available BG in oil-binding capacity, indicating its promising potential for the cosmeceutical industry (Vaithanomsat et al., 2022). *Ganoderma lucidum* G2 spore (GLS) oils extracted by Soxhlet (SLE) and microwave-assisted extraction (MAE) showed distinct properties. MAE-extracted oil had higher oxidative stability, increased unsaturated fatty acids, and more triterpenoids, while SLE-extracted oil demonstrated greater antioxidant activity. Both oils were non-toxic to Caco-2 cells, suggesting MAE-extracted GLS oil as a potential health supplement (Senphan et al., 2024). The sensory profile of *G. lucidum* was characterized to address challenges of bitterness and undesirable flavors. Fresh, dried, and extract forms were analyzed, showing earthy and mushroomy notes as dominant flavors. The study achieved high encapsulation efficiencies for key components like flavonoids and polysaccharides using encapsulation with 32.75% maltodextrin, 42.25% gum Arabic, and 25% modified starch. Gas chromatography electronic nose (GC-E-Nose) analysis detected ten primary flavor compounds, and encapsulation effectively reduced off-flavors. This method enhances the suitability of *G. lucidum* for incorporating instant beverages and other functional foods (Chuensun et al., 2024). *Ganoderma lucidum* (Lingzhi) powders from strains MG2 and G2 were evaluated for nutritional quality. The powders were prepared using water extraction and spray drying, with or without maltodextrin. Both strains provided good energy sources, rich in carbohydrates, proteins, and minerals, with strain MG2 yielding higher germanium content than G2. Maltodextrin addition also influenced the composition of the powders (Vijitrotai et al., 2023).

Five *Ganoderma* species (*G. colossus, G. neojaponicum, G. cattienensis, G. lucidum*, and *G. applanatum*) from Vietnamese National Parks, along with three strains from Europe and Siberia, were analyzed for their morphology, cultural characteristics, and chemical constituents. Valuable compounds such as fatty alcohols and acids, identified through gas chromatography, have potential applications in food supplements, drug delivery, and biodiesel (Tsivileva et al., 2016). The Hmong ethnic group in Lao PDR relies extensively on medicinal plants, including *Ganoderma*, for primary healthcare, with knowledge passed orally and kept within families. A study documented 333 medicinal species, highlighting their use for gastrointestinal, gynecological, and skin conditions, although knowledge transmission remains vulnerable due to economic and cultural factors (Dubost et al., 2019). In a study, 35 (16.2%) of Thai participants used *Ganoderma*, with 6 using homemade products, 17 using registered dietary supplements, 5 using registered herbal medicines, and 8 using undetermined products, all of which were identified as *G. lucidum* (Chotipanich et al., 2019). **Table 9** outlines the nutritional and health benefits of *Ganoderma* species found in the Lower Mekong Basin.

**Table 9:** Nutritional and health benefits of *Ganoderma* species found in the Lower Mekong Basin.

Species	Bioactive compounds	Key findings	Reference
*Ganoderma applanatum*	Fatty alcohols, fatty acids	Contains valuable compounds with potential applications in food supplements, drug delivery, and biodiesel.	Tsivileva et al. (2016)
*G. cattienensis*			Tsivileva et al. (2016)
*G. colossus*			Tsivileva et al. (2016)
*G. lucidum*	β-glucans	Highlighted the presence of β-glucans, supporting its use as a health supplement.	Boonyanuphap and Hansawasdi (2011)
	Extracts (unspecified bioactive compounds)	Found that extracts could help prevent calcium oxalate stone formation, benefiting kidney health.	Pinitsoontorn et al. (2012)
	Crushed *G. lucidum* (unspecified bioactive compounds)	Demonstrated that crushed *G. lucidum* added to smoked fish sausages increased nutritional value and retarded lipid oxidation, showcasing its role in food preservation.	Wannasupchue et al. (2011)
	β-glucan (BG), protein	Extracted β-glucan (BG) for cosmeceutical applications. The extract showed anti-tyrosinase, antioxidation, and moderate anti-collagenase, anti-elastase, and anti-hyaluronidase effects, with no skin irritation.	Vaithanomsat et al. (2022)
	Triterpenoids, unsaturated fatty acids	MAE-extracted GLS oil showed higher oxidative stability, increased unsaturated fatty acids, and more triterpenoids, while SLE-extracted oil demonstrated greater antioxidant activity. Both oils were non-toxic.	Senphan et al. (2024)
	Flavonoids, polysaccharides	Encapsulation with maltodextrin, gum Arabic, and modified starch achieved high encapsulation efficiencies, reducing off-flavors and enhancing suitability for functional foods.	Chuensun et al. (2024)
	Carbohydrates, proteins, minerals, Germanium	Powders from strains MG2 and G2 were rich in nutrients, with strain MG2 yielding higher germanium content. Maltodextrin addition influenced powder composition.	Vijitrotai et al. (2023)
*G. neojaponicum*	Fatty alcohols, fatty acids	contains valuable compounds with potential applications in food supplements, drug delivery, and biodiesel.	Tsivileva et al. (2016)
*Ganoderma* spp.	Various medicinal compounds (unspecified)	Hmong ethnic group in Lao PDR uses *Ganoderma* and other medicinal plants for gastrointestinal, gynecological, and skin conditions. Knowledge transmission is vulnerable due to economic and cultural factors.	Dubost et al. (2019)
*Ganoderma* spp.	Extracts (unspecified bioactive compounds)	35 Thai participants used *Ganoderma* products, including homemade, registered dietary supplements, and herbal medicines, all identified as *G. lucidum*.	Chotipanich et al. (2019)

### 6.9 Enzyme production and industrial applications

Laccase enzymes have diverse industrial applications due to their catalytic oxidation abilities (Asad et al., 2024; Scheibel et al., 2024; Wang et al., 2025). These multicopper oxidases are essential for lignin degradation, textile dye decolorization, bioremediation, and bioactive compound synthesis (Deng et al., 2024; Liu et al., 2024; Tayar et al., 2025). Their utility in wastewater treatment, pulp and paper processing, and pharmaceuticals has been extensively explored (Hanafiah et al., 2024; Hultberg and Golovko, 2024; Pandey and Gupta, 2024). Given their eco-friendly nature and adaptability, laccases serve as valuable biocatalysts in industrial processes.

*Ganoderma* species also play a significant role in environmental monitoring due to their ability to bioaccumulate heavy metals, radionuclides, and pollutants, making them effective bioindicators of contamination (Vyas and Kulshrestha, 2023; Xu et al., 2024; Song et al., 2025). Studies have demonstrated that *G. lucidum* can absorb and retain cesium-137, lead, and cadmium, aiding in pollution assessment (Ipeaiyeda et al., 2020; Jin et al., 2023; El-Baz et al., 2024). In addition, their enzymatic activity, particularly laccase production, contributes to biodegradation and bioremediation, further highlighting their ecological significance.

Studies like Chuwech and Rakariyatham (2014) observed that *G. australe* produced cellulase during fermentation, suggesting potential applications in enzyme production. Khammuang (2009) and Punnapayak et al. (2009) highlighted the laccase activity of *G. lucidum*, which has applications in both industrial dye decolorization and environmental cleanup efforts, demonstrating its broad industrial utility. *Ganoderma lucidum* cultivated in Vietnam was found to contain 137Cs at a concentration of approximately 20 Bq/kg fresh weight, likely accumulated directly from the atmosphere rather than the substrata. While this indicates potential environmental exposure, it also suggests that *G. lucidum* could serve as a bioindicator for monitoring radioactive contamination (Van and Le Duy, 1991). *Ganoderma* has proven industrial applications, particularly in enzyme production. Laccase from *G. lucidum* Chaaim-001 BCU effectively degraded 16 polycyclic aromatic hydrocarbons (PAHs). Complete degradation of anthracene occurred with or without a redox mediator. With the mediator, degradation rates for benzo[a]pyrene, fluorene, acenaphthene, acenaphthylene, and benzo[a]anthracene reached up to 100%, 98.6%, 95.4%, 90.1%, and 85.3%, respectively. In the absence of the mediator, the degradation rates decreased to 71.71%, 62.9%, 80.49%, 85.85%, and 9.14%, respectively. Compared to *Trametes versicolor*, *G. lucidum* laccase demonstrated higher degradation efficiency, particularly in the absence of the mediator. This could be due to the unique structural and catalytic properties of the laccase enzyme from *G. lucidum*, which might allow for more efficient interactions with the PAHs without the need for a redox mediator. These findings highlight its potential for bioremediation of PAHs (Punnapayak et al., 2009). Laccase from *Ganoderma* sp., isolated in northeast Thailand, was purified and characterized for its enzymatic properties. Among three strains tested, *Ganoderma* sp. 03 showed the highest laccase activity when cultured with rice bran and husk. The enzyme, with a molecular weight of 39.81 kDa, had optimal activity at pH 3.5 and 40–55°C, and showed best stability at 30°C. Laccase activity was strongly inhibited by DTT and p-coumaric acid, and reduced by most metal ions except ZnSO_4_. These results highlight the enzyme's potential for industrial and environmental applications (Sarnthima et al., 2022). In Vietnam, *Ganoderma* is valued for medicinal use, but its classification often relies on inaccurate morphological traits. Using ITS-based DNA barcoding, 10 commercial genotypes were analyzed, revealing genetic diversity with distances ranging from 0.000 to 0.047. Seven samples were reclassified as *G. lingzhi* and three as *G. lucidum*. ITS barcoding provides a precise and reliable method for species identification, reducing misclassification that could impact product consistency and efficacy. Accurate classification ensures quality control in commercial *Ganoderma* products, helping to standardize medicinal properties and maintain consumer confidence in the industry (Viết Thế et al., 2019). *Ganoderma lucidum* offers high nutritional value and health benefits but is highly perishable due to its short shelf life. To enhance its value, a process for canning white *G. lucidum* was developed, focusing on preserving total phenolic content and antioxidant activity. Optimal conditions were cooking for 4 min at 90°C with 5 g/L of salt. The resulting canned *G. lucidum* can serve as a functional food (Minh et al., 2019). Quantifying triterpenoids in *G. lucidum* mycelium is challenging due to low concentrations and matrix complexity. A study developed an HR-QTOF-MRM method to quantify seven target triterpenoids, which outperformed the QQQ-MRM method by providing lower detection limits, better reproducibility, and higher accuracy (84%–99% accuracy vs. 69%–114%). Recovery rates for HR-QTOF-MRM ranged from 80% to 117%. Triterpenoid concentrations ranged from 0.06 to 6.72 mg/g in the fruiting body and 0.0009–0.01 mg/g in the mycelium. The HR-QTOF-MRM method improves sensitivity and precision for trace triterpenoid analysis in complex samples (Kaewnarin et al., 2021). *Ganoderma lucidum* extract (GE), rich in ergosterol, flavonoids, and triterpenoids, shows strong antioxidant activity but requires intestinal-targeted delivery. This study developed multilayer microcapsules using sodium alginate (SA) as the primary layer and chitosan (CS) to create SA-CS polyelectrolyte layers for GE encapsulation. The SA-CS layers enhanced controlled release in intestinal conditions (82.15 ± 3.99%) and protected GE from acid, bile, trypsin, and heat, extending its shelf life. This microencapsulation system effectively preserves the active antioxidant compounds in GE for improved delivery and stability (Petwattanapha et al., 2024).

Laccase production in *Ganoderma* sp. KU-Alk4 was influenced by glucose concentration, regulating isozyme expression with molecular masses of 53–112 kDa. A Box–Behnken factorial design optimized culture conditions, increasing laccase activity 12-fold to 240 U/ml. The isozymes exhibited high thermal stability, retaining full activity at 60°C for 1h and optimal activity at pH 3.5. These findings highlight *Ganoderma* sp. KU-Alk4 as a promising source of laccase for industrial applications (Teerapatsakul et al., 2007a, b). **Table 10** explores the enzyme production and industrial applications of *Ganoderma* species found in the Lower Mekong Basin.

**Table 10:** Enzyme production and industrial applications.

Ganoderma species	Application	Reference
*Ganoderma australe*	Cellulase production during fermentation	Chuwech and Rakariyatham (2014)
*G. lucidum*	Laccase activity for dye decolorization & environmental cleanup	Khammuang (2009), Punnapayak et al. (2009)
	Bio-indicator for radioactive contamination (137Cs accumulation)	Van and Le Duy (1991)
	Laccase degradation of PAHs (higher efficiency than *T. versicolor*)	Punnapayak et al. (2009)
	ITS-based DNA barcoding for species identification	Viết Thế et al. (2019)
	Canning process development for functional food	Minh et al. (2019)
	HR-QTOF-MRM method for triterpenoid quantification	Kaewnarin et al. (2021)
	Microencapsulation for antioxidant stability and intestinal delivery	Petwattanapha et al. (2024)
*Ganoderma* sp. KU-Alk4	Optimized laccase production for industrial use	Teerapatsakul et al. (2007a, b)

## 7 Bioremediation

In a study of 18 wood-rotting fungi isolated from Thailand, 5 strains were found to produce mycogenic crystals when grown on media amended with zinc, copper, cadmium, and lead salts. Notably, *Ganoderma aff. steyaertanum* was identified for the first time for its ability to transform heavy metals into metal oxalates. These fungi were capable of converting zinc sulfate into zinc oxalate, copper sulfate into copper oxalate, cadmium sulfate into cadmium oxalate, and lead nitrate into lead oxalate. The results suggest that wood-rotting fungi, including *Ganoderma aff. steyaertanum*, can be used for the detoxification of heavy metal pollution through precipitation as metal oxalates (Kaewdoung et al., 2016). Four wood-decaying fungi—*Polyporus retirugis*, *Trametes* spp., *Lentinus* spp., and *Ganoderma* spp.—were screened for their ability to tolerate and solubilize molybdenum trioxide (MoO_3_). *Polyporus retirugis* and *Lentinus* spp. exhibited the highest solubilization efficiency, producing clear zones over 40 mm on molybdenum-supplemented agar. All strains demonstrated high metal tolerance, although increasing MoO_3_ concentrations reduced mycelial biomass. These findings suggest that these fungi could be effective agents for enhancing molybdate ion bioavailability in fertilizers containing molybdenum compounds (Sutjaritvorakul and Chutipaijit, 2017). *Ganoderma* species, including *Ganoderma* sp. 2, were assessed for decolorizing POME, with moderate efficiency observed. *Ganoderma* sp. 2 showed notable decolorization but was less effective than *Trametes elegans*. Manganese peroxidase activity peaked at 36.03 U/L, while laccase and lignin peroxidase had minimal activity. Biosorption tests with mycelial biomass resulted in a 12.5% color reduction, suggesting limited further decolorization potential through biomass. These results underscore the environmental application potential of *Ganoderma* (Ridtibud et al., 2024). **Table 11** examines the bioremediation potential of *Ganoderma* species in the Lower Mekong Basin.

**Table 11:** Bioremediation potential of *Ganoderma* species in the Lower Mekong Basin.

Species	Bioactive compounds/process	Key findings	Reference
*Ganoderma* aff. *steyaertanum*	Metal oxalates (zinc oxalate, copper oxalate, cadmium oxalate, lead oxalate)	Ability to transform heavy metals into metal oxalates. Capable of detoxifying heavy metal pollution through precipitation as metal oxalates.	Kaewdoung et al. (2016)
*Ganoderma* spp.	Molybdenum trioxide (MoO_3_) solubilization	Demonstrated high metal tolerance but lower solubilization efficiency compared to *P. retirugis* and *Lentinus* spp. Potential for enhancing molybdate ion bioavailability in fertilizers.	Sutjaritvorakul and Chutipaijit (2017)
*Ganoderma* sp.2	Manganese peroxidase, laccase, lignin peroxidase	Showed moderate decolorization efficiency for palm oil mill effluent, with manganese peroxidase activity peaking at 36.03 U/L. Limited biosorption potential for further decolorization.	Ridtibud et al. (2024)

### 7.1 Extraction methods

*Ganoderma lucidum* spores contain abundant bioactive compounds, but their hard sporoderm limits absorption. Traditional spore-breaking methods are costly and inefficient. A study used *Lactobacillus plantarum* fermentation to break down the sporoderm, achieving full breakdown by day 5. This low-cost method enables the production of beneficial fermented juices with *G. lucidum* spores, with further research needed on the breakdown mechanism (Chaiyasut et al., 2010). Polysaccharides from Vietnamese *G. lucidum* spores were extracted using enzymatic, microwave, and ultrasonic methods. The highest yield was achieved with enzymatic extraction using a 2.3% cellulase concentration, 150 min of treatment, and a temperature of 50°C. The complex method, combining preheating, enzyme treatment, ultrasonic, and microwave treatment, resulted in the highest polysaccharide yield of 7.1971% (Mai, 2018) a study optimized ethanol-modified SC-CO₂ extraction for triterpenoids from *Ganoderma lucidum*. Optimal conditions (380 bar, 7% ethanol, 60°C) yielded 1.49g/100g, outperforming traditional methods. The process followed a second-order kinetic model, confirming SC-CO₂ as an efficient extraction technique (Phan et al., 2023). In Vietnam, *G. lucidum* grows year-round and is a valuable source of polysaccharides. An optimized enzymatic extraction process, guided by a central composite design, identified ideal conditions: a 1:50 *G. lucidum* i-to-water ratio, 50°C solvent temperature, 0.25% enzyme concentration, and 2.34-h extraction time, yielding 5.24% polysaccharides. This efficient method is well-suited for industrial application (Sao Mai et al., 2015). Soxhlet and MAE methods yielded the highest extraction rates and total phenolic content from broken *G. lucidum* spores. However, MAE was faster than Soxhlet extraction. Ethanol extraction using MAE produced the highest total phenolic content and ferric-reducing antioxidant power (FRAP) value. Ethanol and hexane extract also showed significant metal chelating activity. These results highlight the importance of selecting the right extraction method and solvent to obtain extracts with high antioxidant properties (Senphan et al., 2021). *Ganoderma lucidum* was dried using convection-assisted microwave drying to improve shelf life. Drying rates increased, and drying times decreased with higher air temperatures (50°C–70°C) and microwave powers (200–600 W). Optimal drying (25 min) was achieved at 70°C and 600 W, while the best color retention and rehydration were at 50°C and 200 W. The drying model accurately predicted moisture content (Inla et al., 2023). The impact of different processing techniques on *G. lucidum* G2 spores (GLS) were evaluated. Vibrating and ball milling increased lipid content and bioactive compounds, with vibrating milling achieving the highest sporoderm breakage (98.27%). Both milling methods enhanced DPPH radical scavenging activity and altered GLS structure, increasing crystallinity. Ball milling led to the highest lipid oxidation. FT-IR and x-ray diffraction analyses revealed key compounds and structural changes. These findings provide valuable insights for utilizing GLS in various applications (Sriket et al., 2024). *Ganoderma lucidum* was evaluated for nutrient and antioxidant extraction under various drying and extraction conditions. Optimal drying at 80°C for 1h and 37 min yielded 1.17 mg/g flavonoids and 11.49 mg/g triterpenes. MAE at 800 W for 1.5 min with 65.35% ethanol provided the highest yields, with 13.08 mg/g polysaccharides and 9.15 mg/g triterpenes. MAE was more efficient than other methods, offering time, solvent savings, and high extraction efficiency for dried *G. lucidum* (Chuensun et al., 2021). **Table 12** explains the methods used in the extraction of *Ganoderma* species in the Lower Mekong Basin.

**Table 12:** Extraction methods used for *Ganoderma* species.

Extraction method	Key findings	Reference
Convection-assisted microwave drying	Optimized drying at 70°C & 600 W (25 min), best color at 50°C & 200 W	Inla et al. (2023)
Enzymatic, microwave, ultrasonic	Highest polysaccharide yield (7.1971%) from combined enzyme, ultrasonic, and microwave treatments	Mai (2018)
Fermentation (*Lactobacillus plantarum*)	Achieved full sporoderm breakdown by day 5, enabling low-cost production of fermented juices	Chaiyasut et al. (2010)
Milling (Vibrating, Ball Milling)	Vibrating milling had 98.27% sporoderm breakage, enhanced bioactive compounds, and antioxidant activity	Sriket et al. (2024)
Microwave-assisted extraction (MAE)	800 W for 1.5 min with 65.35% ethanol yielded the highest polysaccharides (13.08 mg/g) & triterpenes (9.15 mg/g)	Chuensun et al. (2021)
Optimized enzymatic extraction	5.24% polysaccharide yield using 1:50 ratio, 50°C, 0.25% enzyme, 2.34-h extraction	Sao Mai et al. (2015)
Soxhlet and Microwave-assisted extraction	MAE yielded highest total phenolic content, antioxidant activity, and was faster than Soxhlet	Senphan et al. (2021)

## 8 Market potential of *Ganoderma* in Lower Mekong Basin: export and import dynamics

The global *Ganoderma* extract market was valued at approximately USD 2.1 billion in 2023 and is projected to reach around USD 4.5 billion by 2032, growing at a compound annual growth rate (CAGR) of 9.0%. This growth is driven by increasing consumer awareness of its potential health benefits, such as immune system support, anti-cancer properties, liver protection, and cardiovascular health. (Data Bridge Market Research, 2021; *Ganoderma lucidum* Extract Market Research Report, DataIntelo 2023). The *Ganoderma* trade in Southeast Asia, particularly in Vietnam, Cambodia, Laos, and Thailand, is part of a rapidly expanding global market driven by increasing consumer demand for health products derived from medicinal mushrooms. The *Ganoderma* trade in Southeast Asia, particularly in Vietnam, Cambodia, Laos, and Thailand, is part of a rapidly expanding global market driven by increasing consumer demand for health products derived from medicinal mushrooms (Putthapiban et al., 2017). These countries engage in both exports and imports of *Ganoderma*, each contributing uniquely to regional trade while facing distinct challenges and opportunities.

**Figure 2:**
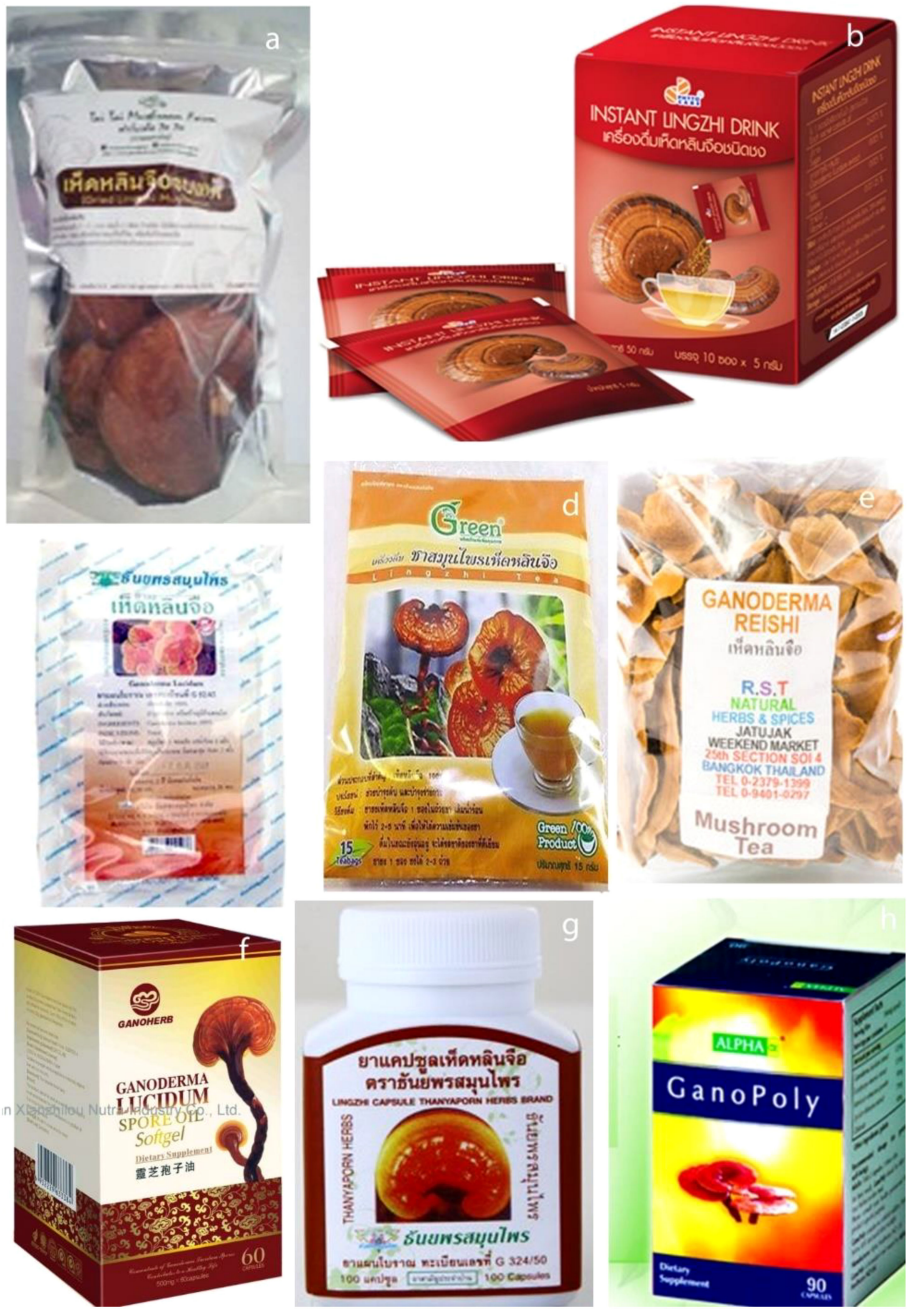
*Ganoderma*-based products **(a)** Dried *Ganoderma lucidum* (https://www.mustthai.com/) **(b)** Instant Lingzhi drink (https://sakasaka.net) **(c)** *Ganoderma lucidum* spore powder (https://thethaiday.com/) **(d)** *Ganoderma* tea (https://thethaiday.com) **(e)** *Ganoderma* tea slices (https://rstspices.com) **(f)** *Ganoderma lucidum* spore oil (www.thaiherbalproducts.com) **(g)** *Ganoderma lucidum* capsules (https://english.thaicare100.com) **(h)** *Ganoderma lucidum* polysaccharides (https://traditionalherbalstore.com).

Thailand is an emerging exporter of *Ganoderma*, with raw mushrooms and processed products like powders, extracts, and capsules being the primary exports (**Figure 2**). The price for raw *Ganoderma* in Thailand is approximately USD 10–14 per kilogram, while processed products are priced at USD 20–35 per kilogram, depending on quality and packaging. Key export destinations include China, Japan, and Europe, with Thailand focusing on organic *Ganoderma* to cater to the growing wellness market (Sornchareon and Methiyothin, 2020). Thailand imports finished *Ganoderma* products, particularly from Vietnam and China. These imports are primarily consumed in the health supplement sector, with increasing demand for natural remedies. Thailand’s agricultural imports in 2023 were significant, with *Ganoderma* products making up a portion of the wellness products sector (Tan, 2023). Vietnam is a leader in the production and export of *Ganoderma* products. The country exports raw mushrooms, powders, and capsules primarily to China, Japan, South Korea, the United States, and Europe. Raw *Ganoderma* is priced at USD 8–15 per kilogram, while processed products such as powder and capsules are priced at USD 20–30 per kilogram. In 2023, Vietnam’s agricultural export value surpassed USD 53 billion, with *Ganoderma* playing a growing role in this sector (Department of Foreign Trade, Thailand, 2023; General Statistics Office of Vietnam, 2024). Despite its strong production capacity, Vietnam imports *Ganoderma* from Cambodia and Laos for processing, which it re-exports in value-added forms. The country also imports some finished *Ganoderma* products from China. Vietnam’s total agricultural imports in 2023 amounted to USD 267 million, which includes raw *Ganoderma* (KPL News, 2023; The Star, 2024).

Cambodia mainly exports raw *Ganoderma* to Vietnam for further processing. The price for fresh *Ganoderma* is around USD 10–12 per kilogram. While the overall export of *Ganoderma* from Cambodia is limited, the country’s agricultural exports increased by 37% in 2023, with Vietnam being a key destination for these niche products (Phnom Penh Post, 2024; The Star, 2024). Cambodia imports processed *Ganoderma* products like capsules and powdered extracts from Vietnam and China. These products are typically priced higher due to the value-added processing in the exporting countries. Demand for *Ganoderma* supplements is growing, especially in urban centers like Phnom Penh and Siem Reap (The Star, 2024). Laos exports raw *Ganoderma* to Vietnam, where they are processed and sold internationally. The price of raw *Ganoderma* is approximately USD 8–10 per kilogram. In 2023, Laos’ total export value to Vietnam reached USD 565 million, including agricultural goods like *Ganoderma* (KPL News, 2023). Laos imports value-added *Ganoderma* products from Vietnam and China, including medicinal capsules and extracts. These products cater to a small but growing domestic market, especially in Vientiane, which is becoming a hub for health-conscious consumers (World Bank, 2023; ASEAN Trade Database, 2024; Data Bridge Market Research, 2022).

The *Ganoderma* market in Southeast Asia, particularly in Vietnam, Cambodia, Laos, and Thailand, faces common challenges, including the lack of standardized production methods, inconsistent quality control, and limited processing infrastructure. These issues, compounded by taxonomic ambiguities that complicate species identification, hinder market growth. However, there are significant opportunities for expansion. Technological advancements in molecular techniques, coupled with sustainable agricultural practices, could address these challenges and enhance production consistency. Regional collaboration, investment in research and development, and the integration of local knowledge with modern science could unlock further potential for the *Ganoderma* market. With the rising global demand for *Ganoderma-*based health products, particularly in immune support and anti-cancer treatments, the traditional significance of this medicinal mushroom adds substantial value to its market potential. By focusing on sustainable cultivation and improving processing capabilities, these countries can strengthen their positions in both regional economies and international trade. **Tables 13**–**15** list *Ganoderma*-based products in Thailand, Vietnam, Laos, and Cambodia, highlighting their diverse applications in health supplements, functional foods, and traditional medicine.

**Table 13:** *Ganoderma* based products available in Thailand.

Product	Importance	Dose	Certification	Thai company
Ganopoly -*Ganoderma* polysaccharides	Increase levels of antibodies in the body, strengthen the body’s immune, reduce the amount of sugar and cholesterol in the blood, help with sleep	N/A	N/A	Alpha Bio-Technology (Thailand) Co., Ltd
Ganopoly 1A	Makes breathing easier, respiratory assistance for the lungs, throat, and trachea, make a strong immune system, reduces allergies and symptoms of asthma, and reduces the risk of inflammation of the pharynx	N/A	N/A	Alpha Bio-Technology (Thailand) Co., Ltd
Ganopoly 3C	Strengthen the immune system, enhance the production of blood cells	N/A	N/A	Alpha Bio-Technology (Thailand) Co., Ltd
Ganopoly the second B	Enhance the activity of the blood circulation system, control the blood sugar and cholesterol levels, reduction of coronary occlusion, reducing the risk of high blood pressure	N/A	N/A	Alpha Bio-Technology (Thailand) Co., Ltd
HepTec	Promote liver function, restoration of cells	N/A	N/A	Alpha Bio-Technology (Thailand) Co., Ltd
Heri-PAG	Improves the performance of the excretory system, relieve constipation	N/A	N/A	Alpha Bio-Technology (Thailand) Co., Ltd
Heri-Poly	Enhance the activities of the digestive system, to relieve gastritis and chronic peptic ulcers	N/A	N/A	Alpha Bio-Technology (Thailand) Co., Ltd
Lingzhi (*Ganoderma lucidum* or Reishi) Herbal mushroom tonic capsules	Reduce the blood sugar and cholesterol levels, stabilize the cell membranes of RBCs	Two capsules 3 times daily before meals.	Thai FDA Registered	Thanyaporn Herbs Co., Ltd
Lingzhi (*Ganoderma lucidum*) capsules (250 mg extract per capsule)	Improves the immune system, helps to protect the liver and helps detoxify the body, dilates arteries and improves oxygen and energy supply to cardiac muscles, lowers blood pressure, and helps diabetes by reducing blood glucose level	3 × per day after meals, two capsule	GMP, HACPP, HALAL	Thanyaporn Herbs Co., Ltd
Lingzhi capsules (320 mg extract per capsule)	Act as antioxidants, for asthma and other respiratory conditions	3 × per day after meals, 2 capsules	Thai FDA Registered	Thanyaporn Herbs Co., Ltd
Lingzhi capsules (400 mg extract per capsule)	Allergies, asthma, diabetes liver disease, cardiovascular problems, hypertension insomnia, anxiety, and depression rheumatoid arthritis and rheumatism, antiaging, strengthening blood circulation for overweight and obesity	2 capsules 3 times per day after meals	GMP, HACPP, HALAL	Thanyaporn Herbs Co., Ltd
Lingzhi tea	Prevents aging by improving the immune system regulating metabolism, and lowering blood pressure	2–3 cups per day	GMP, HACPP, HALAL	Thanyaporn Herbs Co., Ltd
Lingzhi tea	Improves the immune system and dilates arteries thus improving oxygen and energy supply to cardiac muscles, preventing aging	N/A	FDA certified	N/A (family business)
*Ganoderma* Herbal Tea (*G. lucidum* extract)	Boosts immunity, and improves vitality	N/A	N/A	Thai Herbal Co. Ltd.
Coffee With *Ganoderma* & Ginseng Drink	Boosts immunity	N/A	N/A	JK Co-ordinate Co LTD
4-in-1 instant coffee with *Ganoderma*	It acts as an anti-oxidant & enhances the immune system	N/A	N/A	Fancy World Co., Ltd.
Reishi Coffee, Instant coffee mix (*Ganoderma* extract, coffee)	Reduces stress and supports digestion	N/A	N/A	ABC Health Products
Hi-Balanz Reishi Extract 30 Capsules Dietary Supplement (Reishi Extract 300 mg)		To maintain health – 1 capsule daily after a meal, To treat common diseases - 2–4 capsules daily after meals, to treat cancer - 6–10 capsules daily after meals	FDA certified	Hi-Balanz Co Ltd
Extracted juice, *Ganoderma* with honey and lemon	It contains high Vitamins, Calcium, and Fiber, reduces cholesterol, and acts as an anti-oxidant	N/A	GMP, HACCP, ISO, Halal	Foods Planet Co Ltd
Concentrated Reishi mushroom juice	Act as anti-oxidant	N/A		Doi Kham Food Products Co., Ltd
Reishi Extract Powder (Over 50% Polysaccharides, 1% Triterpenoids)	Activates the skin’s immune system, inhibits irritation, protects skin, and smoothes wrinkles, inhibits the release of histamine to prevent allergy, antioxidant, and free radical scavenging activities to prevent DNA damage.	500– 1,000 mg/day	ISO9001, GMP, HALAL, HACCP	AP INTERTRADE
Reishi Skin Cream (*Ganoderma* extract, aloe vera)	Skin rejuvenation, and anti-aging			Thai Natural Care
Lingzhi (*Ganoderma lucidum*, Reishi) Herbal Mushrooms Tonic (250 mg. of *Ganoderma lucidum* per capsule)	Reduce cholesterol, and sugar levels, control blood pressure, stabilize red cell membrane, prevent allergies	Two capsules 3-times per day after meals	N/A	Vatana and P Limited Partnership
Fuji Reishi Cream	Reduce acne and rash, and facial whitening	N/A	GMP	Fuji Cream Dotcom Co., Ltd
Lingzhi Extract plus Cordyceps capsules	N/A	N/A	N/A	Thai Herbal Products Co., Ltd
Lingzhi Extract plus GINSENG Extract capsules	N/A	N/A	N/A	Thai Herbal Products Co., Ltd
Ganolin Lingzhi Extract capsules (Lingzhi Mushroom Extract 300 mg)	N/A	N/A	N/A	Thai Herbal Products Co., Ltd
Instant Lingzhi Drink	N/A	N/A	N/A	Thai Herbal Products Co., Ltd
*Ganoderma* Tonic Drink (*Ganoderma* extract, honey)	Energy booster and liver support	N/A	N/A	Wellness Drinks Co.
Lingzhi (Reishi *Ganoderma lucidum*) Capsules Super Food/Herb	Helps to protect the liver and works in detoxifying the body, dilates arteries, and improves oxygen and energy supply to cardiac muscles	1–2 capsules in the morning and before bed	Thai FDA	Rainbow Brands. Co., Ltd
*Ganoderma* Capsules (100% *Ganoderma lucidum* powder)	Anti-inflammatory, and antioxidant	N/A	N/A	XYZ Biotech Co.

N/A (Not available)

**Table 14:** *Ganoderma* based products available in Vietnam.

Product	Form	Manufacturer/Region	Details
*G. lucidum* Tea	Tea bags	Linasa (Quảng Nam Province)	Made from sustainably farmed local *Ganoderma* mushrooms
*Ganoderma* Extract Powder	Powder	Various local cooperatives	Processed for medicinal use, it is popular for export and local consumption
*Ganoderma* Capsules	Capsules	Pharmaceutical companies (Hanoi)	Marketed for immune-boosting and antioxidant properties
Fresh *Ganoderma* fruiting bodies	Whole mushrooms (raw)	Local Farmers (Quảng Nam)	Sold directly to consumers or enterprises for further processing
*Ganoderma* Tonics	Herbal tonics (liquid)	Traditional Medicine Producers	Combines *Ganoderma* with other herbs used for vitality and immune support.
*Ganoderma* Cosmetics	Skincare products	Various Vietnamese brands	Infused with *Ganoderma* extracts for anti-aging and skin health benefits

**Table 15:** *Ganoderma* based products available in Laos and Cambodia.

Country	Product	Form	Manufacturer/Region	Details
Laos	*Ganoderma* Herbal Teas	Tea bags/loose leaf	Local cooperatives (Luang Prabang, Vientiane)	Combined with local herbs like lemongrass or ginger; it promotes health and relaxation
	*Ganoderma* Capsules	Capsules	Herbal medicine companies (Vientiane)	Used for general well-being and energy boosting
	Fresh *Ganoderma* Mushrooms	Whole mushrooms (raw)	Organic farms in Bolaven Plateau	Sold raw for culinary or medicinal use
Cambodia	*Ganoderma* Herbal Drinks	Liquid tonics	Traditional medicine producers (Phnom Penh)	Blended with Cambodian herbs for vitality and immune support
	*Ganoderma* Extract Powder	Powder	Small-scale cooperatives (Siem Reap)	Primarily used for medicinal purposes
	*Ganoderma* Skincare Products	Creams/serums	Local beauty brands (Phnom Penh, Sihanoukville)	Anti-aging and skin rejuvenation properties with added natural oils

## 9 Future research directions

Building on the current understanding of *Ganoderma* species, future research should explore several critical areas to enhance its medicinal, ecological, and commercial potential. First, there is a need for more comprehensive molecular studies to refine the taxonomy of *Ganoderma*, addressing current challenges in species identification. Advanced techniques like genome sequencing and multi-locus phylogenetic analysis can provide deeper insights into the genetic diversity and evolutionary relationships within the genus, which is essential for accurate classification and quality control.

Secondly, research into sustainable cultivation methods should be prioritized to increase production efficiency while maintaining the ecological balance. Identifying optimal cultivation substrates, environmental factors, and biotechnological interventions for enhancing bioactive compound yield will be crucial for improving the sustainability and profitability of *Ganoderma* farming. Moreover, further investigations into the pharmacological mechanisms of *Ganoderma* bioactive compounds are needed to better understand their therapeutic effects. This includes studying the molecular pathways by which *Ganoderma*-derived compounds exert their immunomodulatory, anticancer, and anti-inflammatory effects, thus advancing the potential for novel drug development.

In parallel, research into the ecological roles of *Ganoderma* species, especially their interactions with soil ecosystems and their potential use in bioremediation and agriculture, can provide insights into their broader environmental significance. Specifically, their capacity to degrade pollutants, including polycyclic aromatic hydrocarbons (PAHs), warrants further exploration to assess their applicability in sustainable environmental practices. Finally, with the growing commercial interest in *Ganoderma*-based products, more emphasis should be placed on standardizing the quality control processes for both cultivation and product development. Establishing uniform criteria for bioactive compound content, consistency in therapeutic effects, and product safety will ensure the reliability of *Ganoderma*-derived products in the global market. By addressing these areas, future research will contribute to the sustainable development of the *Ganoderma* industry, maximize its medicinal benefits, and expand its applications in diverse sectors, including healthcare, agriculture, and environmental protection.

## 10 Conclusion

*Ganoderma* species, particularly *G. lucidum*, hold significant medicinal and economic value in the Lower Mekong Basin, where demand for their bioactive compounds is increasing. ITS-based DNA barcoding has improved species identification, addressing taxonomic challenges, while optimization of cultivation methods enhances production efficiency. Despite their therapeutic potential, challenges such as inconsistent cultivation, sustainability concerns, and quality control persist. To maximize their applications in medicine, agriculture, and industry, standardized cultivation practices and regulatory measures are essential. Strengthening research on *Ganoderma* taxonomy, ecological roles, and commercial viability will position the Lower Mekong Basin as a leader in sustainable *Ganoderma* production, benefiting both local economies and global healthcare.
